# Brain age estimation at tract group level and its association with daily life measures, cardiac risk factors and genetic variants

**DOI:** 10.1038/s41598-021-99153-8

**Published:** 2021-10-18

**Authors:** Ahmed Salih, Ilaria Boscolo Galazzo, Zahra Raisi-Estabragh, Elisa Rauseo, Polyxeni Gkontra, Steffen E. Petersen, Karim Lekadir, André Altmann, Petia Radeva, Gloria Menegaz

**Affiliations:** 1 Department of Computer Science, University of Verona, Verona, Italy; 2 William Harvey Research Institute, NIHR Barts Biomedical Research Centre, Queen Mary University of London, Charterhouse Square, London, EC1M 6BQ UK; 3 Barts Heart Centre, St Bartholomew’s Hospital, Barts Health NHS Trust, West Smithfield, London, EC1A 7BE UK; 4 Departamento de Matemàtiques i Informàtica, University of Barcelona, Barcelona, Spain; 5 Centre for Medical Image Computing (CMIC), Department of Medical Physics and Biomedical Engineering, University College London, London, UK

**Keywords:** Neuroscience, Neural ageing, Developmental biology, Ageing

## Abstract

Brain age can be estimated using different Magnetic Resonance Imaging (MRI) modalities including diffusion MRI. Recent studies demonstrated that white matter (WM) tracts that share the same function might experience similar alterations. Therefore, in this work, we sought to investigate such issue focusing on five WM bundles holding that feature that is Association, Brainstem, Commissural, Limbic and Projection fibers, respectively. For each tract group, we estimated brain age for 15,335 healthy participants from United Kingdom Biobank relying on diffusion MRI data derived endophenotypes, Bayesian ridge regression modeling and 10 fold-cross validation. Furthermore, we estimated brain age for an Ensemble model that gathers all the considered WM bundles. Association analysis was subsequently performed between the estimated brain age delta as resulting from the six models, that is for each tract group as well as for the Ensemble model, and 38 daily life style measures, 14 cardiac risk factors and cardiovascular magnetic resonance imaging features and genetic variants. The Ensemble model that used all tracts from all fiber groups (FG) performed better than other models to estimate brain age. Limbic tracts based model reached the highest accuracy with a Mean Absolute Error (MAE) of 5.08, followed by the Commissural ($\hbox {MAE}=5.23$), Association ($\hbox {MAE}=5.24$), and Projection ($\hbox {MAE}=5.28$) ones. The Brainstem tracts based model was the less accurate achieving a MAE of 5.86. Accordingly, our study suggests that the Limbic tracts experience less brain aging or allows for more accurate estimates compared to other tract groups. Moreover, the results suggest that Limbic tract leads to the largest number of significant associations with daily lifestyle factors than the other tract groups. Lastly, two SNPs were significantly (p value $< 5\hbox {E}{-}8$) associated with brain age delta in the Projection fibers. Those SNPs are mapped to *HIST1H1A* and *SLC17A3* genes.

## Introduction

Aging is a complex process with substantial impact across multiple organ systems, yet to be fully characterised. In the specific case of the brain, previous studies have found evidence of considerable structural alterations of white and grey matter (WM/GM) structures as well as of morphological and functional connectivity changes across different areas^1^. These modifications are associated with distinct aspects of cognitive functions, emotions, and neurodegenerative disorders^2^. Several studies have demonstrated that groups of WM tracts that share the same function experience similar alterations during the life course and in specific brain disorders. In particular, Yang et al.^3^ investigated the association of brain aging with WM integrity and functional connectivity in a group of healthy subjects. Their findings demonstrated that Projection, Association and Commissural fibers were substantially affected by aging resulting in a significant reduction of their WM integrity, while Brainstem tracts were relatively preserved. In another study, Bender et al. compared different diffusion-based indices estimated over Association, Commissural and Projection fibers again in a healthy population^4^, demonstrating a greater microstructural decline over time in the first FG compared to the Commissural and Projection ones, and a differential aging of cerebral WM. Moreover, the tracts that connect frontal and parietal heteromodal cortices have been shown to be more prone to age-related differences than those from projection fibers^5^. In this context, the so-called “lastly maturing, first going out” phenomenon, grounding on previous magnetic resonance imaging (MRI) evidence^4,6,7^, is of great importance. This refers to a mirroring pattern of development and aging of the human brain, where the last regions to develop are degenerating relatively early^6^. In particular, primitive sensorimotor structures encounter the most rapid development and greatest preservation, while more advanced structures (e.g., prefrontal cortex) seem to have slower development and faster decline, leading also to differential developmental trajectories across WM tracts^5–7^. Therefore, existing work suggests differential aging-related changes depending on the specific WM FGs, which might result in diverse patterns of disease and cognitive impairment. However, the determinants of these different alteration patterns have not been adequately investigated so far.

Neuroimaging modalities can be adopted to estimate the so-called brain age which allows monitoring the longitudinal progression of brain during lifecourse. This is defined as the apparent biological age of the brain, when comparing individuals’ data against a population dataset spanning a range of ages^8,9^. The difference between predicted brain age and actual (chronological) age, generally referred to as “predicted age delta” (brain-PAD), is often computed to verify whether a subject’s brain appears younger or older than their chronological age^10^. Indeed, since humans do not experience brain aging at the same rate and pronounced differences possibly related to genetic and environmental factors are present, brain-PAD can be exploited as a novel biomarker to assess brain aging progression in both healthy and diseased populations. Greater brain age (positive brain-PAD) has been associated with increased risk of neurodegenerative diseases, whilst younger brain age (negative or small brain-PAD) correlates with healthy environmental exposures and lifestyle habits^11^. Among these factors, daily lifestyle, physical activity, electronic device use, and sleeping habits have all shown significant effects on brain progress during the lifecourse^9,12^, with smoking and greater alcohol intake frequency closely linked to increased brain-PAD for instance. Similarly, genetic factors also have a crucial role in brain aging.

In a recent study, Jonsson et al.^8^ demonstrated the presence of two single nucleotide polymorphisms (SNPs) significantly associated with brain-PAD by relying on a genome wide association study (GWAS), which were correlated with reduced WM surface area and reduced sulcal width^8^. Other studies identified several SNPs associated with brain-PAD, with the most significant ones located in *MAPT*^12^ and *TMEM106B* genes^13^. These two genes have been shown to be closely associated with frontotemporal dementia^14^, and *MAPT* has also been considered as a model of interaction in Parkinson’s disease between functional disease outcomes and genetic^15^.

Furthermore, there is a growing evidence suggesting complex cross-system interactions between brain and cardiovascular systems^17–19^. Indeed, cardiovascular risk factors (CRFs) have been already associated with poorer cognitive function. Precisely, higher body mass index (BMI) has been linked to poorer performance across multiple cognitive indications including working memory, attention, delayed recall, and category fluency^20^. In addition, other risk factors such as diabetes and hypertension have been associated with unhealthy brain aging, abnormal neuroanatomical alterations, and increasing risk of developing AD^21^. Finally, DeLange et al.^22^ demonstrated that CRFs such as stroke risk score and alcohol intake are associated with older appearing brains. All these elements deserve further investigations to better understand whether they might influence the brain aging processes differently.

In this context, neuroimaging data derived from MRI sequences have demonstrated to provide accurate estimates of the apparent age of individuals’ brains, generally relying on age regression models^23^. Most brain-age models only use T1-weighted structural MRI, reflecting brain volumes. However, the possibility to use complementary modalities mapping different aspects of brain structure and function has opened the way to the estimation of modality-specific brain aging models. In particular, diffusion MRI (dMRI), resting-state/task functional MRI (fMRI) and susceptibility weighted imaging are currently exploited in different studies to extract novel image-derived phenotypes (IDPs) to be used in specific brain-age models, thanks to the new opportunities offered by large-scale multimodal databases such as the UKB^24^. . Statistical methods for modeling brain age using neuroimaging data are generally highly accurate, with MAE of predictions in the range of 4-5 years for most of the studies relying on different regression approaches such as simple linear regression, support vector regression (SVR) and least absolute shrinkage and selection operator (LASSO)^9,11,12,23^.

In addition, most of these previous studies have demonstrated better results when including multimodal neuroimaging data rather than a single modality in the models^22,26,27^. In particular, findings from these multimodality studies suggest that dMRI measures have higher accuracy in predicting brain age compared to those derived from fMRI, SWI or even anatomical images in some cases^1,11^. The diffusion-based features are generally extracted starting from the microstructural maps estimated using different models, such as the diffusion tensor imaging (DTI) and the neurite orientation dispersion and density (NODDI), and then averaging the corresponding values over several WM tracts. Fractional anisotropy (FA) along with indices of diffusivity (mean/axial/radial [MD/AD/RD]) can be estimated from the DTI model, informing on the degree of anisotropy/diffusivity of diffusion process^28^. Conversely, more complex indices are derived from NODDI, a compartmental model where brain microstructure is described in terms of a set of predefined parameters that is neurite orientation dispersion (OD), representing the directional overall coherence of modeled axons, isotropic volume fraction (ISOVF), showing the unhindered water volume fraction, and intracellular volume fraction (ICVF) that represents neuronal density^29–31^. Previous works have demonstrated the importance of DTI and NODDI IDPs for estimating brain age in both healthy and diseased populations^32,33^. Moreover, microstructural patterns have been demonstrated to follow different trajectories in brain aging within WM structures. In particular, FA tends to decrease during aging while MD, AD and RD have the opposite pattern^9,34^.

In this study, we aimed at estimating and comparing diffusion-specific brain ages in a large cohort free from clinically diagnosed neurological disease from the UKB database, relying on dMRI measures of different FGs in order to assess the impact of aging on WM at the tract-group level. Indeed, investigating brain aging for tracts with shared functionality may permit a more accurate assessment compared to brain aging for the whole brain. In addition, for each FG, we evaluated the relationship between brain predicted ages and several factors spanning across different scales, relating in particular to daily lifestyle, health, cardiac measures and genetics to verify whether a differential association might be present in specific WM tracts. This will also allow to identify those factors that can negatively impact brain aging, providing further insights on its complex mechanisms.

## Materials and methods

### Datasets

#### Participants

Data from $\hbox {n} =$ 16,394 participants with complete brain and cardiac MRI assessment were initially downloaded from the UKB database. Of these, 1059 subjects who reported neurological disorders that could directly affect cognitive function were excluded in order to include only people who met criteria for being neurologically intact at the time of scanning. These were identified using the self-reported medical conditions at baseline extracted from detailed questionnaires that the UKB participants had to answer, the relevant ICD-10 code, hospital episode statistics, and algorithmically-defined outcomes. This led to a final group of 15,335 subjects (mean age $54.79 \pm 7.45$, 7277 males, 8058 females). The complete list of conditions and ICD-10 codes used as inclusion/exclusion criteria are available in supplementary table 1.

All the methods were conducted in accordance with the relevant guidelines and regulations and all participants provided informed consent. UKB received ethical approval from the NHS National Research Ethics Service on 17th June 2011 (Ref 11/NW/0382) and extended on 10th May 2016 (Ref 16/NW/0274). More details can be found on the UKB resource page https://biobank.ndph.ox.ac.uk/showcase/catalogs.cgi. The present analyses were conducted under data application number 2964.

#### Brain and cardiac MRI features

The UKB brain imaging protocol was implemented on a 3T Siemens scanner (Skyra, VD13A SP4, Siemens Healthcare, Erlangen, Germany) and included six different sequences, covering structural, diffusion and functional imaging for a total of 35 min scan time. In particular, a multi-shell protocol has been used for dMRI data, with two b-values ($b = 1000$, 2000 s/$\hbox {mm}^2$), a 2-mm isotropic resolution and a multiband acceleration factor of 3. 50 diffusion-encoding directions were acquired per shell, covering a total of 100 distinct directions over the two b-values. Full details on the neuroimaging data can be found at https://biobank.ctsu.ox.ac.uk/crystal/crystal/docs/brain_mri.pdf. Cardiac MRI was performed on a 1.5T Siemens scanner (MAGNETOM Aera, Syngo Platform VD13A, Siemens Healthcare) according to a pre-defined protocol^36,37^. Left and right ventricular (LV, RV) function was assessed using standard long and short axis acquisitions.

#### Genotype data

UKB genotyped genetic data for 488,377 participants were obtained using two genotyping arrays. A small subsets of the participants (49,950) involved in UKB Lung Exome Variant Evaluation (UK BiLEVE) study were genotyped using the Applied Biosystems UK BiLEVE Axiom Array by Affymetrix. Conversely, the majority of the participants (438,427) was genotyped using the closely related Applied Biosystems UKB Axiom Array. More details about genotyping and genotype calling steps can be found in^38^.

### Feature extraction

#### Brain microstructure feature extraction

In the current study, we relied on the IDPs derived centrally by the researchers involved in the UKB project and made available via the data showcase ( https://biobank.ctsu.ox.ac.uk/crystal/index.cgi). Of these, we focused on the 675 dMRI IDPs extracted for each participant using the following pipeline. First, both the diffusion tensor and the NODDI models were fitted to the pre-processed data leading to nine voxelwise microstructural maps, namely FA, MD, axial diffusivity (L1), radial diffusivities (L2, L3) and mode of anisotropy (MO) from DTI, and ICVF, ISOVF, and OD from NODDI. Two sets of measures were used as microstructural features, both obtained from the UKB repository and extracted following two different approaches^24,39^. The first used tract-based spatial statistics (TBSS). Each individual dMRI map was aligned to a standard-space WM tract skeleton and a series of ROIs was then defined as the overlap of this skeleton with 48 standard-space tract masks from the JHU ICBM-DTI-81 atlas^40^. For each skeletonised microstructural index, the mean value was calculated in each region, leading to a total of 432 IDPs (that is 48 ROIs times 9 IDPs). The second relied on probabilistic tractography. A total of 27 major tracts were identified using standard-space start/stop ROI masks defined by AutoPtx toolbox (http://fsl.fmrib.ox.ac.uk/fsl/fslwiki/AutoPtx). The mean value of each DTI/NODDI parameter was calculated across each tract and weighted by the tractography output as in Alfaro et al.^39^ in order to emphasize values in regions most likely to belong to the tract of interest, resulting in a total of 243 IDPs (27 tracts times 9 IDPs). Table 2 in the supplementary material shows these tracts and their FG.

**Figure 1:**
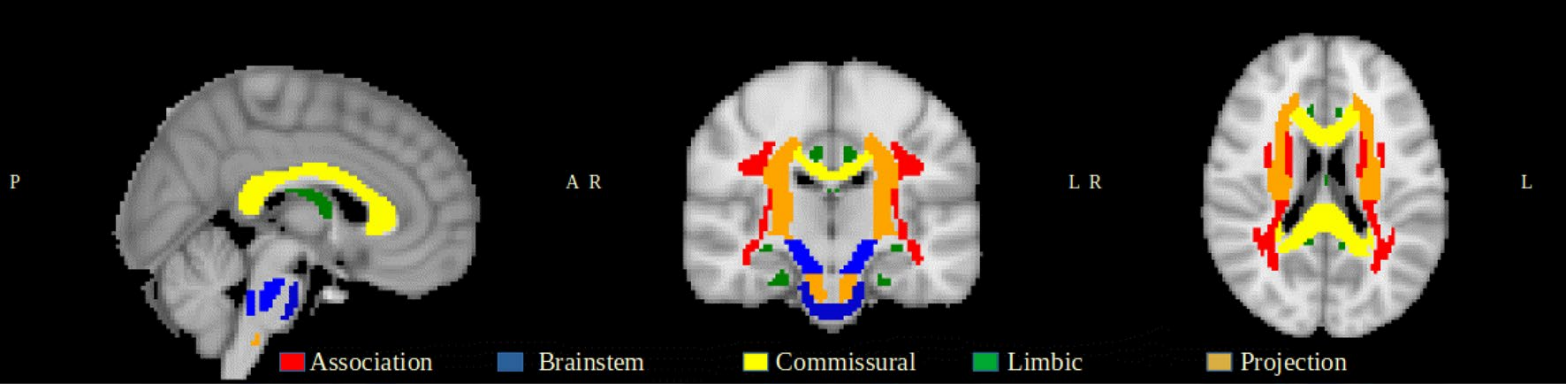
White matter tract groups.

Each ROI and tract was subsequently assigned to one out of five FGs following the fiber tract-based atlas^41^. In particular, the following FG were considered: (1) Association; (cortex-cortex connections); (2) Brainstem; (3) Commissural (left-right hemispheric connection); (4) Limbic; and (5) Projection (cortex-brainstem, cortex–spinal cord and cortex-thalamus connections) fibers. Each FG consisted of a different number of tracts, that is: 22 for Association, 13 for Brainstem, 13 for Commissural, 9 for Limbic, and 18 for Projection. An illustration of these five fiber families is reported in Fig. 1, where the different tracts are depicted in different colors. *Association* fibers interconnect different cortical areas in the same hemisphere^42^. These might be short association fibers that connect adjacent gyri, or long association fibers linking more distant parts of the cerebral cortex. Important examples of this category are the superior/inferior longitudinal fasciculus, inferior fronto-occipital fasciculus, and uncinate fasciculus^43^. *Brainstem* fibers involve the tracts that connect cerebrum to the spinal cord and cerebellum^44^. These includes the corticospinal tract, the posterior column-medial lemniscus pathway and the spinothalamic tract. *Commissural* fibers interconnect corresponding cortical regions of the two hemispheres and are mainly represented by the corpus callosum and anterior commissure^42^. *Limbic* fibers involve structure in both sides of thalamus^45^. Fornix is one of the main vital tract of this system^46^, alongside the Cingulum bundle that connects parietal, frontal and temporal lobe^47^. Finally, *Projection* fibers connect cortical areas with deep nuclei, cerebellum, brainstem, and spinal cord^48^. Corticospinal and corona radiata tracts are the two main examples for this category^49^. By subsequently assigning each ROI and tract to the respective FG, summary IDP values could be finally derived by averaging across ROIs and tracts, respectively, in each FG. In this way, the whole set of IDPs were assigned to each FG resulting in a total of 18 IDPs (9 from the ROI-based and 9 from the tract-based analyses).

#### Cardiovascular feature extraction

Cardiovascular Magnetic Resonance (CMR) data were analysed using an automated pipeline^50^. The extracted cardiovascular indices included measures of LV and RV structure and function. Specifically, the indices derived for the LV were end-diastolic volume (LVEDV), end-systolic volume (LVESV), stroke volume (LVSV) and mass (LVM). The RV indices included stroke volume (RVSV), end-diastolic volume (RVEDV) and end-systolic volume (RVESV) were considered. LV and RV volumes are markers of cardiac remodelling, from these stroke volume may be derived as a measure of ventricular function. LVM is an independent risk predictor in clinical cohorts and an indicator of heart aging in population cohorts. To correct for variation in CMR metrics related to body size, these measures were indexed to body surface area (calculated as per Du Bois formula)^51^. As an additional measure of arterial health in a larger sample, we considered arterial stiffness index (ASI) derived from finger plethysmography^52^. ASI was measured at the baseline UKB visit using the PulseTrace PCA2 (CareFusion, USA) device according to a pre-defined protocol, UKB Arterial Pulse-Wave Velocity (2011) that is available at https://biobank.ndph.ox.ac.uk/showcase/showcase/docs/Pulsewave.pdf. Outliers were removed from the ASI variable using a 1.5 interquartile range (IQR) rule. Finally, CRFs included hypertension, diabetes, deprivation (reported in UKB as the Townsend index), body surface area (BSA), BMI and exercise level.

#### Lifestyle features

Regarding daily life measures, 38 variables were available in the UKB database at baseline. The lifestyle and environment measures included seven categories that are: physical activity (7 measures), sun exposure (2 measures), electronic devise use (2 measures), smoking (2 measures), sleeping habits (5 measures), alcohol (3 measures) and diet (17 measures). All the used variables are available in supplementary Table 3.

### Brain age estimation

All the analyses performed in our study were carried out using Python 3.8.5 and Scikit-learn version 0.23.2. A tract-based healthy aging model was defined for each of the five FG, using the corresponding 18 dMRI IDPs as neuroimaging predictors and the chronological age as dependent variable. To account for the different measurement scales, the features were normalized to zero mean and unit variance^9^. Sex, education level, height and volumetric scaling from T1-weighted head image to standard space were used as covariates because they could be statistically associated with the outcome variable, as previously reported in similar studies^9,11,53^. A Bayesian ridge regression model was run in combination with a 10-fold cross-validation, where the data samples were randomly assigned into ten equal-sized groups. For each group of left out data, the other $90\%$ of subjects were used to estimate the model parameters which were then applied to this additional group for validation. The performance of each model was assessed using MAE and Coefficient of Determination ($\hbox {R}^{2}$).

Several studies have revealed a proportional bias in brain age estimation related to regression model dilution, leading to a significant age-dependency between delta age and chronological age^54,55^ that needs to be statistically corrected. In this study, we adopted the method proposed by Beheshti et al.^54^ which entailed calculating the regression line between brain-PAD and chronological age in the training set:

1 $$\begin{aligned} D = \alpha * \Omega + \beta \end{aligned}$$

where *D* is the brain-PAD from training data, $\alpha$ and $\beta$ represent the slope and the intercept of the linear regression model, and $\Omega$ is the corresponding chronological age. Then, these intercept and slope values were used to correct the predicted brain age in the validation set set as follows:

2 $$\begin{aligned} CPBA = Predicted\, Brain\, Age -(\alpha * \Omega + \beta ) \end{aligned}$$

where CPBA represents the corrected predicted brain age (bias-free). After bias correction, the brain-PAD was calculated as the actual age subtracted from the brain-predicted age. Pearson correlation was calculated between actual and predicted brain age as well as actual age and brain-PAD, both before and after the bias correction steps. An Ensemble tract-based aging model was finally defined using the overall 90 dMRI IDPs (18 from each FG), and the same analyses detailed above were implemented.

### Association analysis

In order to highlight the role of the different variables to model brain age, the association between delta values as resulting from the five FG models and a set of imaging/non-imaging variables was assessed using linear regression model. This included the corresponding 18 dMRI IDPs, 14 CRFs/CMR measures, and 38 daily life measures. In addition, the same analyses were performed for the brain-PAD values derived from the Ensemble model, with the only difference being represented by the associations with the whole set of 90 IDPs for the dMRI part (rather than 18 only). In all models, the brain-PAD represented the outcome measure, while the feature of interest was the independent variable alongside all the above mentioned covariates plus age^9,11^. The resulting p-values were Bonferroni-corrected for multiple comparisons at $\hbox {alpha}= 0.05$^56^, assuming that each model is independent from the others. The p-values were multiplied by the number of tests performed in each analysis, 18 for the associations with the IDPs in each of the five FG models and 90 for the Ensemble model. The association was considered significant if the corrected p-value was less then 0.05.

Of note, Cook’s distance was used to identify potential influential observations before performing the association analyses. In particular, a subject was removed if the Cook’s distance was greater than 3 times the mean distance of all the subjects^12^. The association between genetic variants and brain-PAD values as resulting from each model was also conducted. The quality control steps on SNPs included Minor allele frequency (MAF) thresholding at 0.01, missing rate less than 0.02 and Hardy–Weinberg equilibrium p value $>=1E{-}6$. Quality control on samples ensured that all participants had genotyping rate $> 0.98$, heterozygosity rate within 3 standard deviation, matched genetic/reported gender and were of European ancestry (according to both genetic ethnicity based on principal component analyses and self-reported ethnicity). Related samples were removed based on kinship coefficient $> 0.1$. The quality control steps resulted in 574,492 autosomal SNPs and 12,364 subjects for the GWAS analyses. Thereafter, linear regression was performed using PLINK^57^ and adjusted for education, gender, age, volumetric scaling from T1-weighted head image to standard space, and 40 genetic principal components of ancestry. For each GWAS analysis, FUMA^58^ was used to map the significant SNPs to genes based on positional mapping and eQTL. Using FUMA and GTEx (https://gtexportal.org/home/), we also identified Expression quantitative trait loci (eQTL) to take advantage of gene expression. Finally, we looked at UKB genetic data (http://big.stats.ox.ac.uk/)^59^ to find association between the significant SNPs and other phenotypes.

## Results

### Brain age estimation

The impact of aging was separately assessed in terms of MAE and $\hbox {R}^{2}$ values after fitting the five considered multivariate linear FG-based models plus the Ensemble one. The mean and standard deviation of such values across a 10-fold cross-validation were reported in order to probe the reliability of the estimation. Results are summarized in Table 1 where the columns 2–6 correspond to the five FG, that is, Association, Brainstem, Commissural, Limbic and Projection fibers, and the last column reports the results for the FG Ensemble. In the table, the Pearson correlation coefficient between the actual age and the predicted age before (CAPB) and after (CAPA) correction, the actual age and the brain-PAD before (CADB) and after (CADA) correction are also reported in the last four rows.

As it can be observed, the performance is quite uniform across FG, with the exception of the Brainstem group especially regarding the $R^2$ value that is the lowest. The best MAE was obtained for the tract Ensemble model followed by the Limbic FG, which also corresponds to the highest $R^2$. The last four rows prove that the age-bias was successfully removed.

**Table 1:** Performance of the five FG-based models plus the Ensemble one to estimate brain age in terms of MAE and $R^2$. The last four rows provide the CAPB, CAPA, CADB and CADA, respectively. The best performing model is identified by star symbol. The last four rows provide the CAPB, CAPA, CADB and CADA, respectively. The best performing model is identified by star symbol.

Matrices	Association	Brainstem	Commissural	Limbic	Projection	Ensemble
Mean $\hbox {R}^{2}$	0.26	0.11	0.26	0.29	0.25	$\star$ 0.42
STDV $\hbox {R}^{2}$	0.02	0.01	0.01	0.02	0.03	0.015
Mean MAE	5.24	5.86	5.23	5.08	5.28	$\star$ 4.55
STDV MAE	0.1	0.09	0.11	0.12	0.13	0.08
CAPB	0.51	0.33	0.51	0.54	0.5	0.65
CAPA	0.91	0.95	0.91	0.9	0.91	0.89
CADB	$-$ 0.85	$-$ 0.94	$-$ 0.86	$-$ 0.83	$-$0.86	$-$0.75
CADA	$-$ 0.001	$-$ 0.003	$-$ 0.001	$-$ 0.001	$-$ 0.001	$-$ 0.006

### Association studies

#### IDPs association with brain-PAD

**Figure 2:**
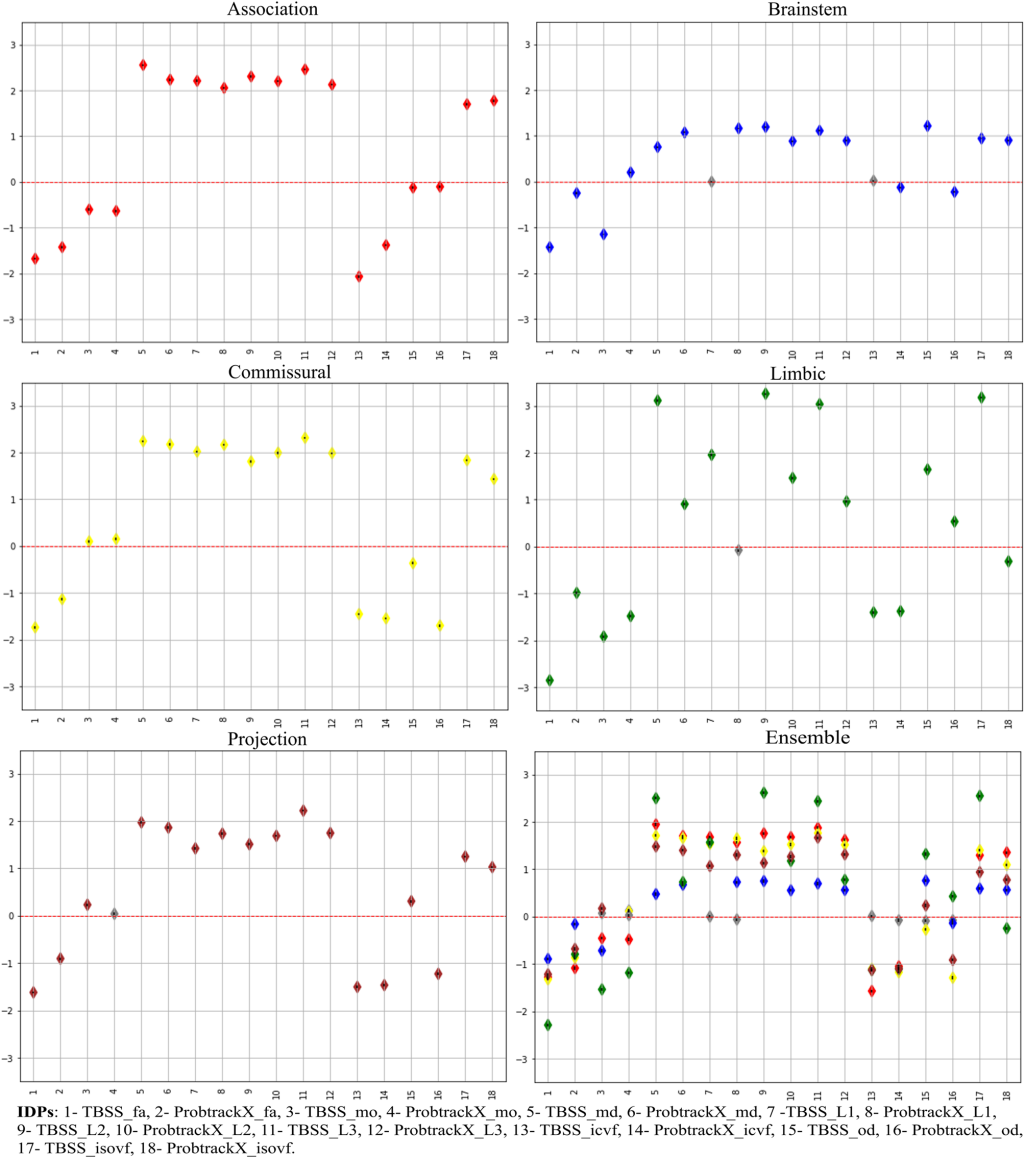
Association of the IDPs and brain-PAD for the different models. For each model, the numbers on the x-axis represents the order of the different IDPs summarised in the legend, while the regression coefficient (the diamond shape represents the beta coefficient) values are reported in the y-axis along with their standard error (the small black dot inside the diamond shape). Grey color indicates non-significant association.

Linear regression results describing the relationships between the bias-adjusted brain-PAD values for the five FG-based models and each microstructural IDPs are illustrated in Fig. 2. In addition, results for the Ensemble model are also reported, including in this case the associations with 90 dMRI IDPs rather than 18 as in the case of the previous five FG models. The coefficient value are unitless because we standardized the IDPs and brain-PAD before performing the analysis. The coefficient value refers to how many standard deviations a dependent variable (brain-PAD) will change per standard deviation increase in the independent variable (individual IDPs) A highly similar association pattern is apparent across FG, though higher variability was observed for the Limbic FG. More specifically, all the IDPs were significantly associated with brain-PAD in Association and Commissural groups, while few associations did not reach significance in the other three tract groups, that is: mean L1 and mean ICVF in Brainstem, weighted mean L1 in Limbic and weighted mean MO in Projection fibers. Considering the different imaging variables, anisotropy (FA, ICVF, OD and MO and respective weighted versions) and diffusivity (MD, L1, L2, L3, ISOVF and weighted versions) indices led to associations of opposite direction, as expected, in almost all the cases. More precisely, FA and weighted FA showed a significant negative association with brain-PAD in all five groups, while MD/weighted MD presented the opposite pattern and appeared to more strongly contribute to modelling the outcome in all cases. Similarly, increased L1, L2 and L3 plus their weighted versions were associated with increased the brain-PAD for all groups, except L1 and weighted L1 in Brainstem and Limbic FGs, respectively. Finally, the weaker associations were observed for MO and weighted MO in all models. Consistently with what above, for NODDI-based measures, the diffusivity index ISOVF was positively associated with the brain-PAD in all cases. A slightly different pattern was observed for OD and its weighted version across the FG, that presents a higher variability. OD is positively associated with the brain-PAD, as expected, in Brainstem and Limbic fibers, though not in the weighted version, and has a different pattern in the other three groups, with a prevalence of a negative association of the weighted version. The association between the IDPs and the brain-PAD was also assessed FG-wise relying on the Ensemble model, revealing that the pattern was preserved though with slightly different values. In particular, the association was slightly reduced with respect to the values that were obtained for FG-specific brain-PADs. Please refer to table 4 in the supplementary for more details regarding the association.

#### CRFs and vascular measures association with brain-PAD

**Figure 3:**
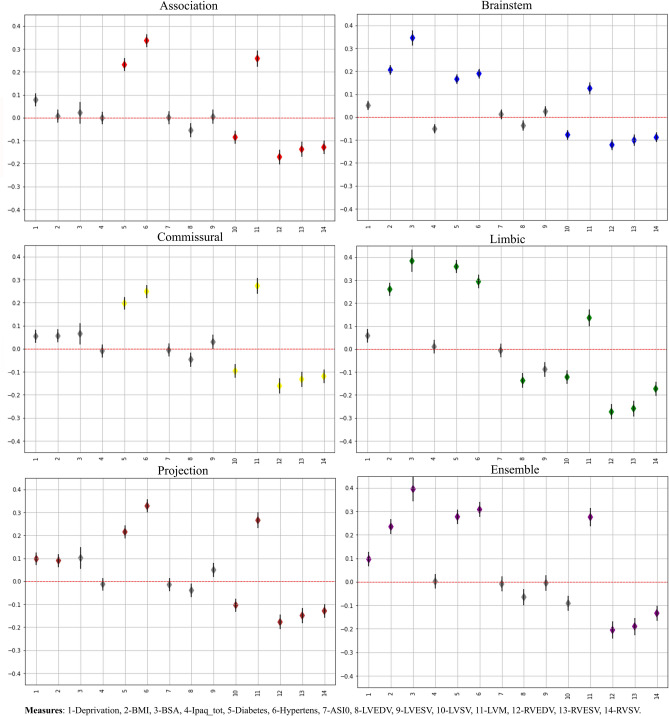
Association of the CMR measures, CRFs and brain-PAD. For each model, the numbers on the x-axis represents the order of the different CMR and CRFs measures summarised in the legend, while the regression coefficient (the diamond shape represents the beta coefficient) values are reported in the y-axis along with their standard error. Grey color indicates non-significant association.

Figure 3 reports the results of the linear regression analyses between the bias-adjusted brain-PAD values and the CRFs/CMR measures, revealing consistent patterns across the five FG models. In all conditions, several measures were significantly associated with PAD after multiple comparison correction, in particular increased brain-PAD was associated with a diagnosis of diabetes, hypertension and increased LVM, as well as with reduced LVSV/RVSV and RVEDV/RVESV. Greater BMI was also associated with increased brain-PAD in three out of five models (Projection, Brainstem and Limbic), with the last two FGs also showing a positive relationship between delta and BSA. Of note, the model based on Limbic fibers presented the highest number of significant associations and the direction of the relationships was consistent in all the five FC-based models. The same trend was observed for the Ensemble model. These associations followed the same pattern compared to the other five FG models. More precisely, the association results were closer to those found for the the Brainstem and Limbic FG, especially in eight out of 14 measures. Please refer to table 5 in the supplementary for more details regarding the association.

#### Lifestyle association with brain-PAD

**Figure 4:**
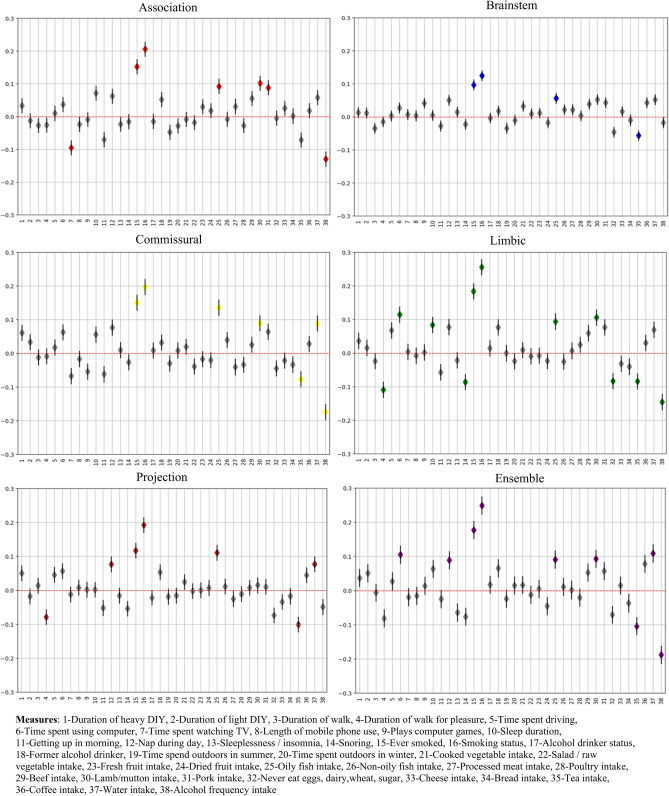
Association of daily lifestyle measures and brain-PAD. For each model, the numbers on the x-axis represents the order of the daily lifestyle measures summarised in the legend, while the regression coefficient (the diamond shape represents the beta coefficient) values are reported in the y-axis along with their standard error. Grey color indicates non-significant association. A unique color was assigned to each group measures(e.g physical activity).

Figure 4 reports the results of the linear regressions between the bias-adjusted the brain-PAD values and the daily life measures in each of the five FG-based models plus the Ensemble one. Consistent patterns were visible across the FG.

The highest number of significant associations was observed for the Limbic tracts, while only four measures survived for the Brainstem group, though in agreement with the others. In all cases, increased brain-PAD was associated with ever smoked, smoking status, greater oily fish intake, and tea intake (except for Association fibers). In addition, increased brain-PAD values from Association, Commissural and Limbic fibers were associated with greater lamb/mutton intake and greater frequency of alcohol intake. Duration of walk for pleasure had a positive impact on brain age, being associated with reduced brain-PAD values in both Limbic and Projection fiber models, while increased brain-PAD was associated with water intake in Commisural and Projection FG models. Finally, seven additional daily life measures, including time spent watching TV or using computer and sleep duration, presented only selective associations in one of the models (4 for Limbic, 2 for Association and 1 for Projection). Please refer to table 6 in the supplementary for more details regarding the association. The coefficient value for all those are significantly associated with brain-PAD in FG is small (less than 0.3) indicating small effect.

#### Association between SNPs and brain-PAD

**Figure 5:**
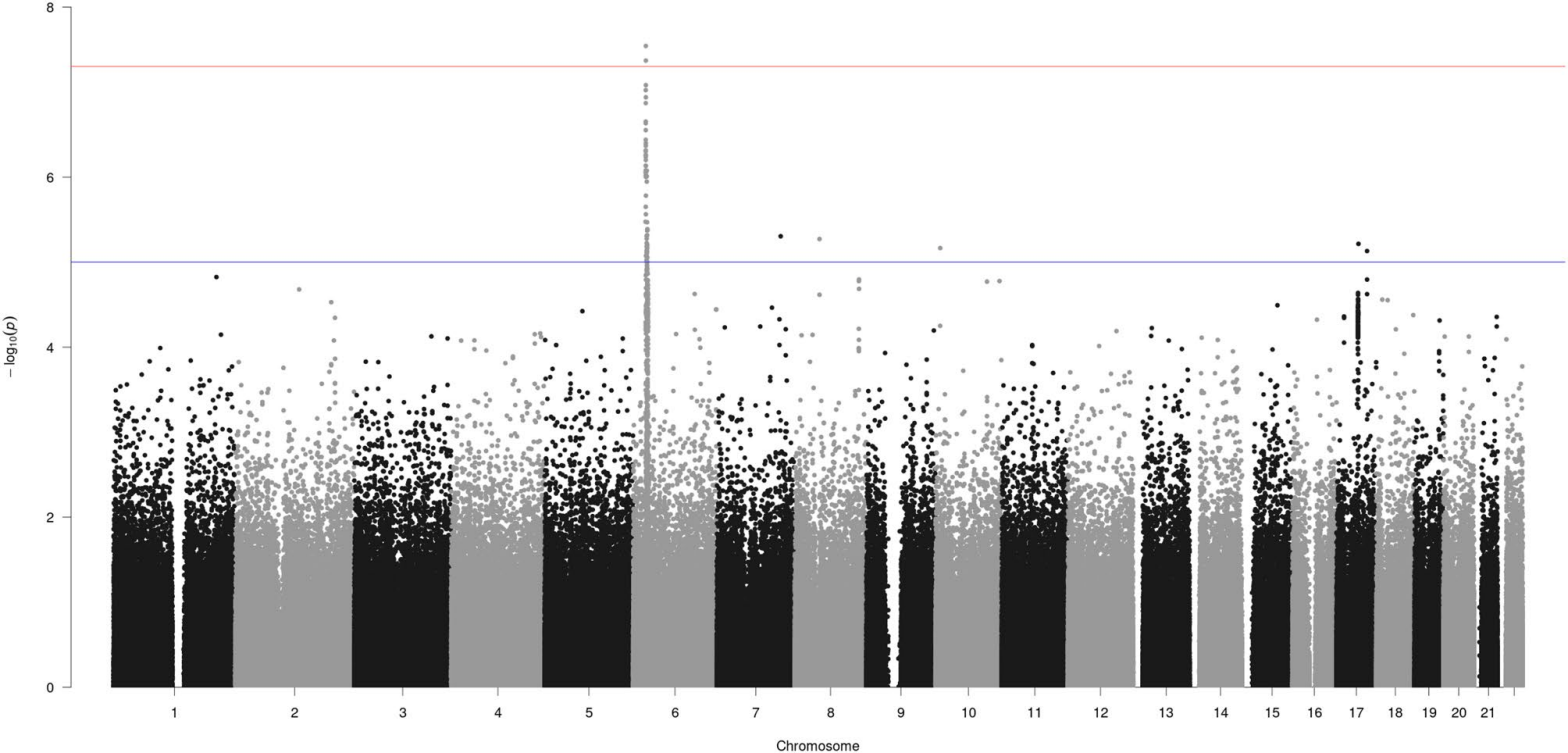
Manhattan plot reporting the association results between SNPs and brain-PAD in Projection FG. The red line indicates the GWAS threshold on p value (i.e.,5E−8), while the blue line indicates the suggestive threshold of $\hbox {p}=5\hbox {E}{-}5$.

Two SNPs located on chromosome 6 showed significant associations ($\hbox {p} < 5\hbox {E}{-}08$) with brain-PAD values in the Projection FG, namely rs1045537 ($\hbox {p} = 2.87\hbox {E}{-}08$) and rs16891334 ($\hbox {p} = 4.268\hbox {E}{-}08$). Figure 5 illustrates the Manhattan plot showing the association between the SNPs in all chromosomes and brain-PAD from the Projection FG.

Moreover, the Manhattan plots for the other FG and the Ensemble model were also reported (Appendix, Figures 1 to 5).

The leading SNP (rs1045537) was mapped to *BTN3A* cluster *(BTN3A1 to BTN3A3)*, *SCGN, SLC17A* cluster *(SLC17A1 to SLC17A4)*, *HIST1H1A* group of genes based on FUMA results using positional mapping and eQTL based on GTEx database (version 8 54 and 8 30) and general tissue types. In addition, it is significantly associated significantly with forced vital capacity, BMI, headache and coeliac disease in UKB cohort.

## Discussion

This study focuses on providing a holistic view on the endogenous end exogenous factors shaping brain aging as expressed by brain microstructural features of specific WM tracts, providing hints for the multiscale and multifactorial analysis of the system ‘human being’. The challenge being to link heterogeneous information living at different scales, this work takes a step in that direction by linking microscopic (genes), mesoscopic (dMRI IDPs), macroscopic (cardiovascular IDPs) and behavioral (lifestyle) measures through their respective association to the brain age picture provided by dMRI. After investigating the potential of microstructural measures derived from dMRI in estimating brain-PAD relying on five different FG, the associations of neuroimaging, genetic and cardiovascular IDPs with brain-PAD were assessed and, as a final step, lifestyle and behavioral measures were also considered. The rest of this section will be dedicated to the discussion of the results as well as of the potential consistency of the observed associations across scales while referring to the existing literature.

The estimated brain-PAD was minimized by the Ensemble model, gathering the whole set of $18 \times 5$ microstructural features, leading to the minimum MAE (4.55 years) and the maximum $R^2$ (0.42). On the other end, Brainstem FG led to worst performance, with the highest value for the MAE (5.86 years with std = 0.01) and the minimum for $R^2$ (0.11). The Brainstem FG, including the midbrain, pons, and medulla, involves structures with complex WM pathways and GM nuclei that are concentrated in a small area. Intricate Brainstem circuitries are difficult to capture using conventional dMRI measures such as DTI, with the consequence that both the tractography and the estimation of microstructural indices are prone to errors^60^.

Among the other single FG-based models, the Limbic one provided the best MAE (5.08 with $\hbox {std} = 0.02$) and $R^2$ (0.29), closely followed by the others (Association-, Commissural- and Projection-based FG models) showing a similar pattern. The Ensemble model relying on all available IDPs provided the best results compared to single FG-based models, suggesting that the inclusion of multiple features from different WM FG could better depict the modulations related to brain aging and therefore lead to more accurate estimates.

However, this would impeed to disambiguate the impact of the aging process on the different FGs, that is the main objective of this work. The association of PAD with the dMRI IDPs revealed the path these measures follow in brain aging. Based on the corrected p value, their association with PAD wes significant in all FGs apart from very few cases. Fractional anisotropy (FA) and ICVF were reduced in all tract groups while L1, L2, L3, MD, ISOVF increased with advancing age. This is in agreement with the expectation since they are consistent with neuronal loss as discussed in [ADD REFS]. The contribution of OD and MO was relatively inconsistent among tract groups featuring an increment in some FGs and a decrement in others. The pattern was similar for the Association, Commissural and Projection FGs, as well as for the Ensemble model. The results for Limbic tract showed a different pattern compared to other FGs. Overall, our results are inline with what have been published before in terms of the direction these IDPs follow in brain aging, as reported, for instance, in Smith et al.^9^. Their results indicate that FA and ICVF decrease with aging while L1, L2, L3, MD and ISOVF increase. In addition, they showed that the dMRI features are among of those most relevant for the estimation of brain age in Fornix irrespectively of the sex. Our results are consistent with these findings since as the IDPs from the Limbic FG, which is dominant in the Fornix tracts, were on the top of the list of relevant features to model brain age in the Ensemble model. Another interpretation for such results could be that brain age is more accurately estimated in these regions than other regions which result in reduced error (MAE). In addition, the diffusion indices in the Limbic tracts, specially in the Fornix, might be very sensitive to aging and indicate an atrophy of the tract rather than alterations in WM microstructure^16^.

Most of CRFs and CMR measures led to significant associations with brain-PAD in different FGs. The direction of the association was shared by all tract groups. Brain-PAD in the Limbic FG was significantly associated (5 positively and 5 negatively) with most of these measures. Brainstem FG brain-PAD was significantly associated with 4 measures negatively and 5 positively. Brain-PAD in Ensemble model was significantly and positively associated with 6 measures and significantly and negatively associated with 3 measures. Positive associations was two times compared to negative with brain-PAD in the case when all IDPs were used to model brain age, while association and commissural seem less affected. Among these measures, diabetes, hypertension and LVM were positively associated with brain-PAD in all tract groups while RVEDV, RVESV and RVSV were negatively associated with brain-PAD in all tract groups. The other measures showed inconsistent association with brain-PAD across different tract groups. Body mass index and diabetes were positively associated with brain-PAD which indicates induced acceleration in brain aging. Based on these results, people who suffer from diabetes might experience accelerate brain aging by about half a year, consistently with^11^ reporting an acceleration of about 2 years. The difference might be related to the features preprocessing and normalization steps. The body mass index has been associated with risk of developing neurodegeneration or cognitive decline. Increasing in adiposity in overweight and obese individuals might alter the WM volume that causes faster brain aging up to 10 years^61^. Cardiac index is significantly associated with brain aging even for healthy people. People who present a lower cardiac index or least pumping blood rate appeared almost 2 years older than those having highest cardiac index^21^. Moreover, low cardiac index might be an indication of increase risk of brain disorders. In^62^ they have concluded that low cardiac index increase the risk of incident Dementia and AD. Our results demonstrate novel associations between accelerated brain-PAD and vascular risk factors. However, as we do not account for potential co-existence of multiple risk factors we cannot conclude independent associations with individual risk factors. More detailed examination of these relationships including accounting for possible confounding and evaluation of mediating mechanisms is warranted, although beyond the scope of the present work.

Regarding daily lifestyle factors and activities, 11 measures had significant associations with limbic tracts. Among them, 5 measures were negatively associated with brain-PAD meaning that these factors might slow down and preserve from brain aging. Brainstem tracts were significantly associated with only 4 measures, out of which three were positive (indicating accelerated brain aging) and one was negative (indicating delaied brain aging). Association, Commissural and Projection FG showed close results of 7 significant associations for each one of them. For the Ensemble model, 9 measures were significantly associated with brain-PAD, and mostly were positively associated. Among all these measures, smoking statues and alcohol frequency intake was significant in all cases (apart from alcohol frequency intake in Brainstem). Alcohol frequency intake was negatively associated with brain-PAD which indicates acceleration in brain aging. This has been confirmed before in other studies. Of note, alcohol frequency intake was coded as lower value means higher intake. In^63^, alcohol intake history was negatively associated with WM volume specially in corpus callosum. In addition, alcohol frequency intake was associated with deleterious in WM tracts cause atrophy in Ensemble and regional brain^64^. Our findings are inline with previous studies and this was observed in most tract groups. Two variables were considered to define smoking status based on data available in UKB these included ever smoked (UKB ID 20160) and smoking status (UKB ID 20116). Smoking is associated positively with brain-PAD suggesting that smoking has a negative impact on brain aging. It should be noted that smoking habits is being associated with different alterations in both WM/GM. Moreover, smoking duration linked with reduced total volume of WM . It is also associated with reduction in FA in the cingulate gyrus^65^. Lamb/mutton intake was also positively associated with brain-PAD in some tract groups. Low red meat intake has been associated with better cognitive function^66^. In addition, limited consumption of red meat might reduce risk of AD, slow cognitive decline and reduce AD biomarker such as atrophy^67^.Time spent using computer and sleep duration were positively associated with brain-PAD in Limbic fibers causing acceleration in brain aging. Finally, duration of walk for pleasure was negatively associated pointing to a healthy brain aging as walking stimulates blood circulations and exposition to the sun light.

The association of SNPs and brain-PAD in different FGs led to the identification of one significant locus with leading SNP rs1045537 ($\hbox {p} < 5 \times 10^{-8}$) in Projection fibers. Significant association was observed between rs1045537 SNP and an eQTL of *BTN3A2* in heart left ventricle, basal ganglia, Frontal Cortex and Cortex. *BTN3A2* gene has been identified as a potential risk gene for schizophrenia^68,69^. The SNP is also significantly associated with malabsorption/coeliac disease, body mass index and headache. *HIST1H1A* gene was associated with DNA methylation at early AD stages^70^. *SCGN* gene was identified as one of the most common psychostimulants in brain-wide targets^71^. *SLC17A2* is one of the solute carrier family that is membrane protein and transporter. It was associated with neurodegenerative disorders because of its important role in the recovery of neurotransmitters^72^.

Estimating brain age for a specific region within brain or using different modes of structural and functional change were proposed before to detect the alterations in brain functions and structures in both healthy and diseased populations. Kaufmann et al.^73^ estimated brain age using features from frontal, occipital, temporal, cingulate, parietal, insula, or cerebellar–subcortical regions. They found that the brain-PAD was increased in dementia and multiple sclerosis when the model estimated brain age using features only from cerebellar–subcortical while the largest effect was observed in the frontal lobe in schizophrenia. Smith et al.^16^ estimated brain age using 62 modes representing distinct patterns of structural and functional brain alteration and distinct patterns of association with genetics, cognition, lifestyle, disease and physical measures. They suggested that modelling of distinct pattern of brain alterations would provide more biologically meaningful biomarkers to detect brain aging than one single homogeneous process.

To conclude, in this study we propose to detect the disparity in the alterations of WM FGs through life-course using brain age. Results suggest that brain PAD holds the potential as an aging biomarker. Moreover, it shows which FGs are more prone to aging than others which could be further investigated and exploited to estimate an aging-driven risk factor and an alert for cognitive decline and brain disorders related to the regions in each FG. In addition, we explored the association between daily life style, CRFs and genetic variations and their effects on each FG as well as on the Ensemble model gathering all the considered tracts. Such kind of associations can be employed to examine the influence of environment and genetic factors to shape and control the aging process and related alterations at a FG level, providing a more localized information than the one obtained using the whole WM. Overall, consistent results were obtained regarding the associations in different FGs. Some FGs showed similar pattern for the different considered associations. One of the main contributions of the study shows which FGs are more affected by the aging process as reflected by the considered IDPs. Furthermore, we were able to show that the Limbic FG plays a prominent role in driving brain aging. In addition, brainstem FG observed to age faster and lest affected by the used measures in the analysis, precisely with daily life styles and activities This could be explained that brainstem might age faster as it is more involved in many body functions. Benarroch^74^ reported that Brainstem involves tracts that are critically associated with the control of the cardiovascular function, respiration, arousal and wake-sleep cycle. In that matter, brainstem tracts are more prone to alterations due to direct association with body organs. These findings suggest that further research is required to obtain a more comprehensive understanding of the role of Limbic and Brainstem tracts in brain aging and their association with both body functions and environmental exposures.

## Supplementary Information

Supplementary Figures.

Supplementary Table 1.

Supplementary Table 2.

Supplementary Table 3.

Supplementary Table 4.

Supplementary Table 5.

Supplementary Table 6.
