# Supplementary material for: Brain age estimation at tract group level and its association with daily life measures, cardiac risk factors and genetic variants
Source: Sci Rep. 2021 Oct 18;11:20563. doi: 10.1038/s41598-021-99153-8 (PMC8523533; doi:10.1038/s41598-021-99153-8)

**Figure 1** – Manhattan plot reporting the association results between SNPs and brain-PAD in Association FG. The red line indicates the GWAS threshold on p-value (i.e.,5E-8), while the blue line indicates the suggestive threshold of p=5E-5.


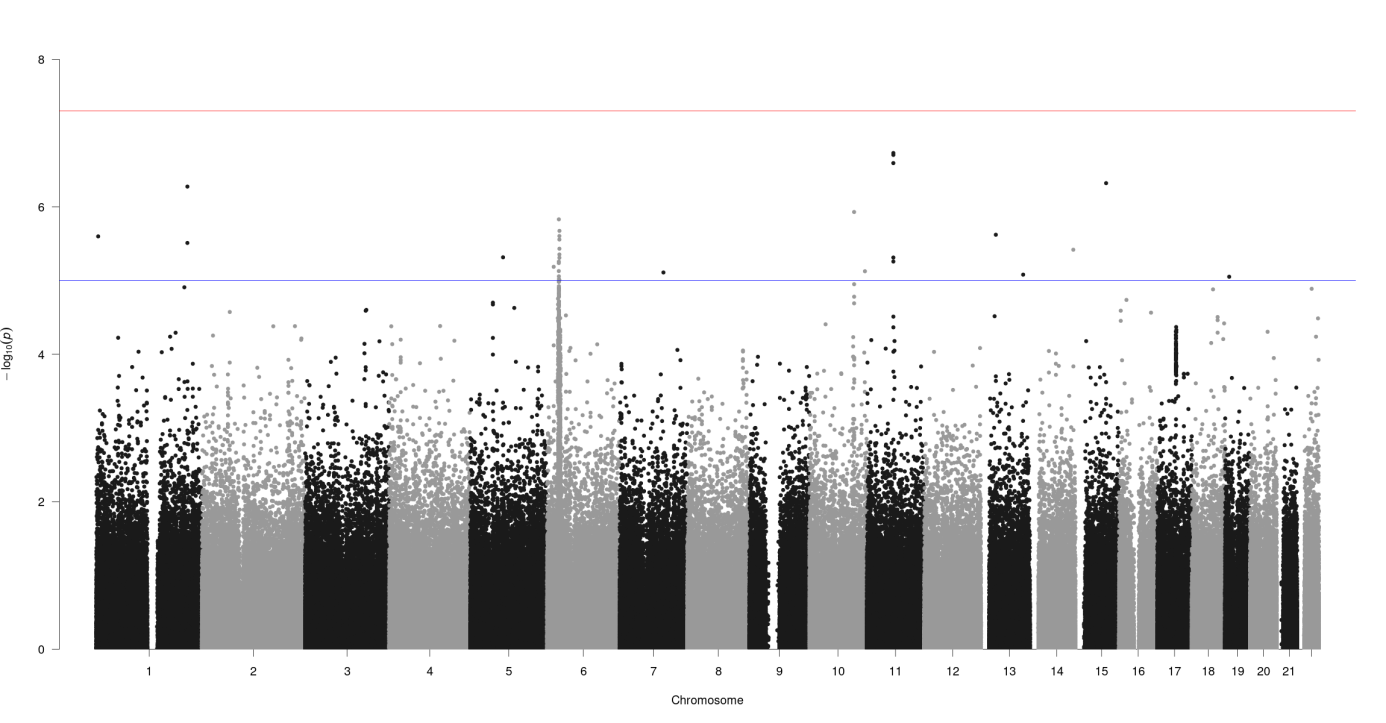


**Figure 2** – Manhattan plot reporting the association results between SNPs and brain-PAD in Brainstem FG. The red line indicates the GWAS threshold on p-value (i.e.,5E-8), while the blue line indicates the suggestive threshold of p=5E-5.


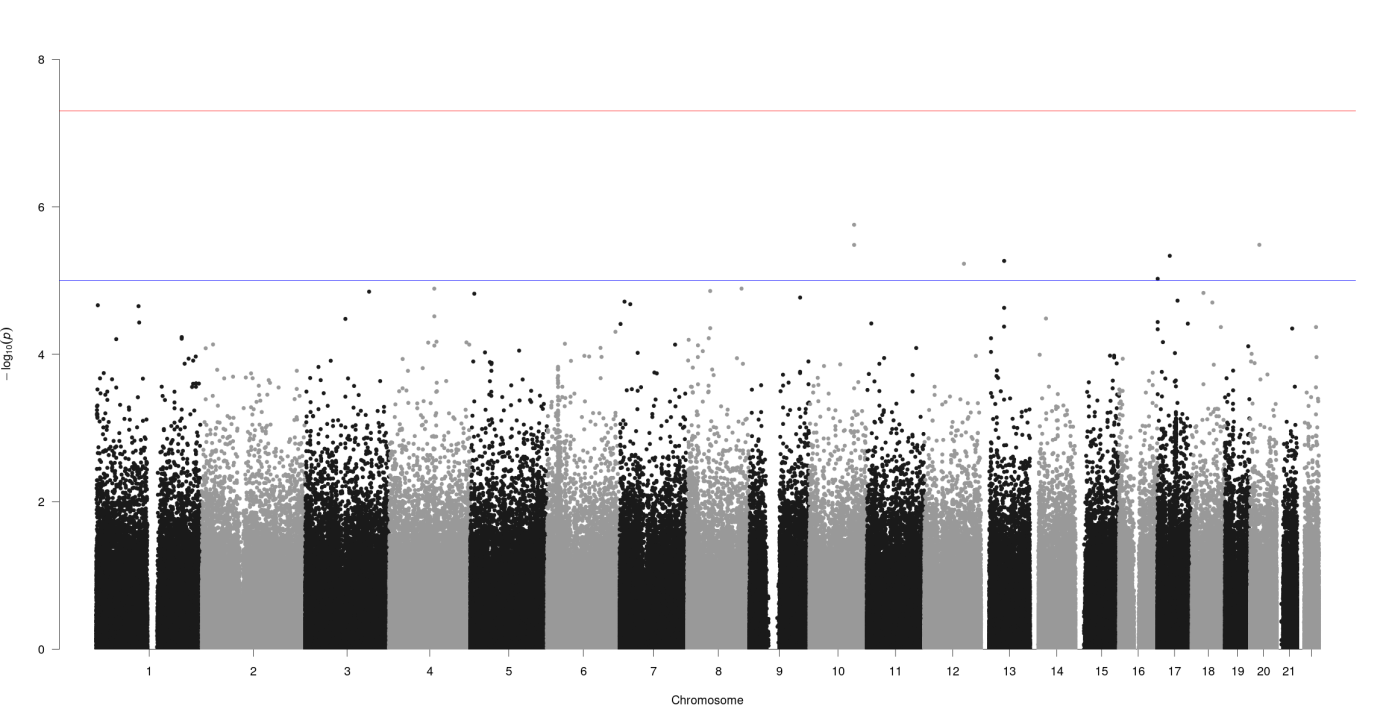


**Figure 3** – Manhattan plot reporting the association results between SNPs and brain-PAD in Commissural FG. The red line indicates the GWAS threshold on p-value (i.e.,5E-8), while the blue line indicates the suggestive threshold of p=5E-5.


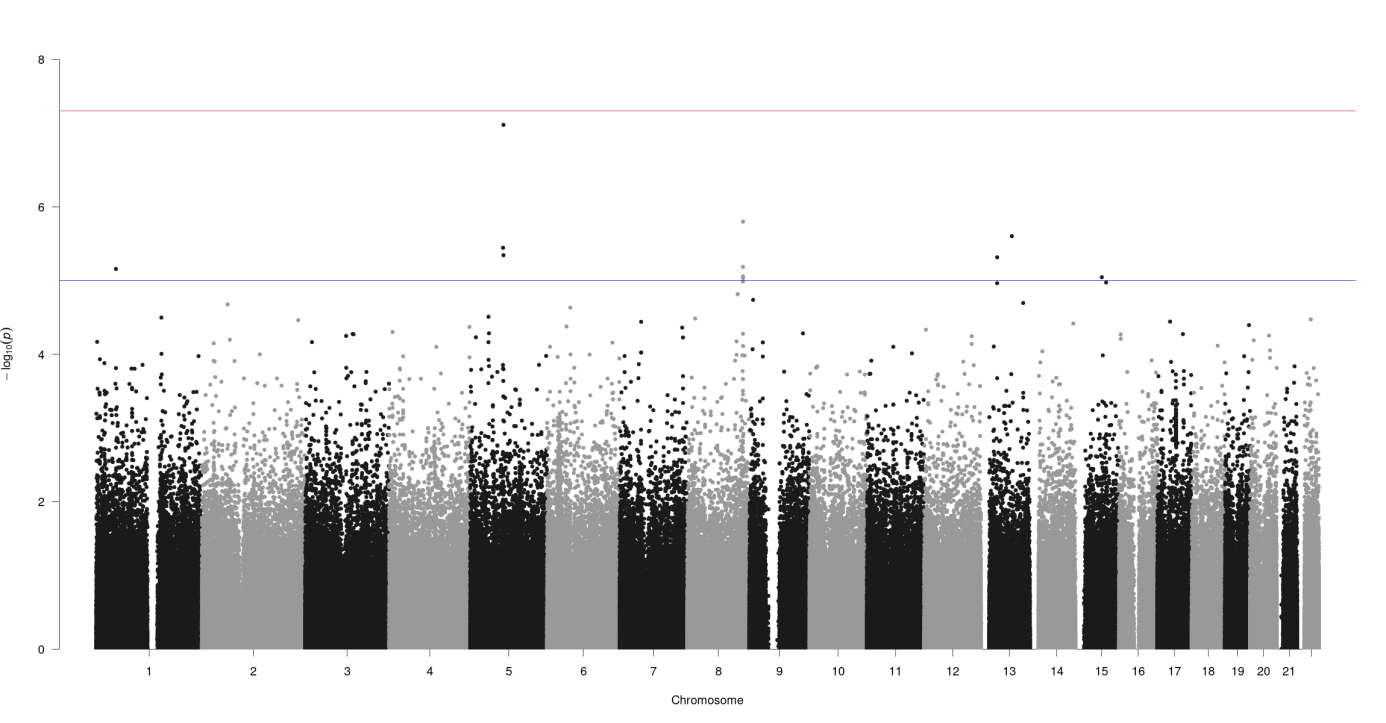


**Figure 4** – Manhattan plot reporting the association results between SNPs and brain-PAD in Limbic FG. The red line indicates the GWAS threshold on p-value (i.e.,5E-8), while the blue line indicates the suggestive threshold of p=5E-5.


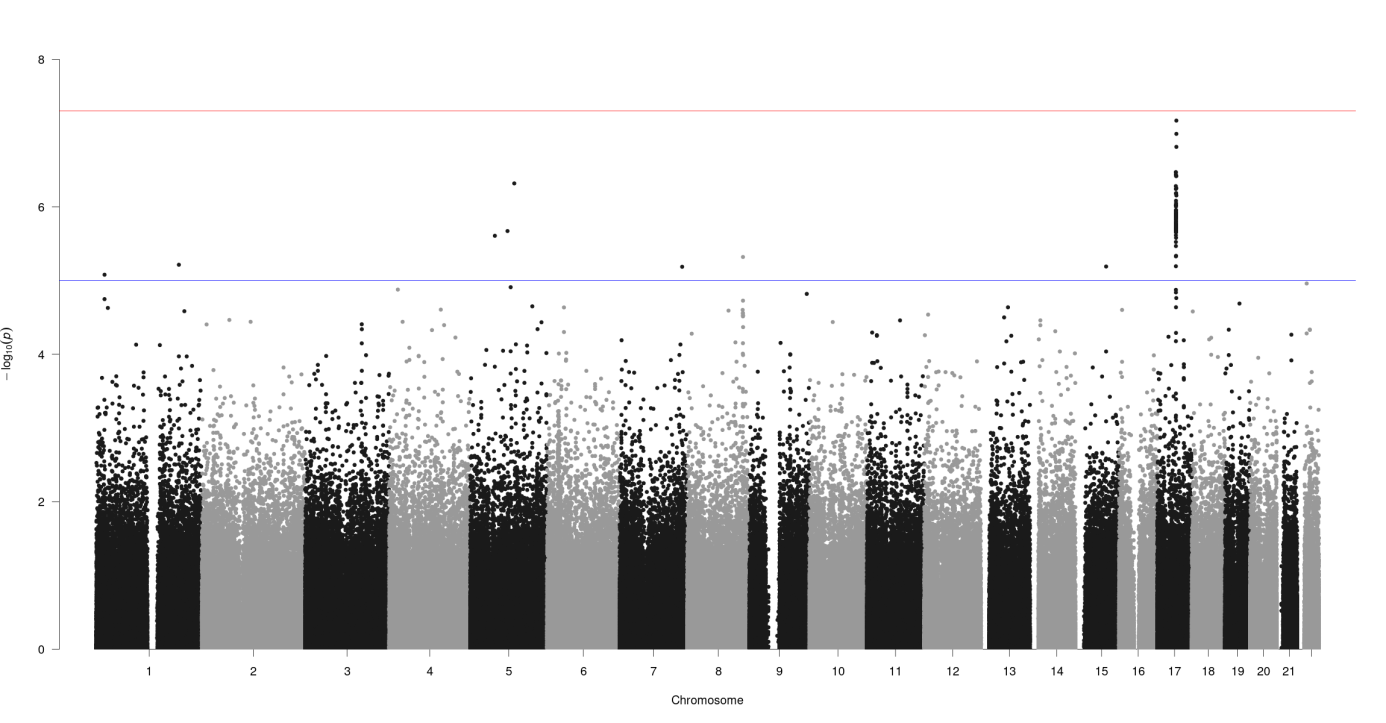


**Figure 5** – Manhattan plot reporting the association results between SNPs and brain-PAD in Ensemble model. The red line indicates the GWAS threshold on p-value (i.e.,5E-8), while the blue line indicates the suggestive threshold of p=5E-5.


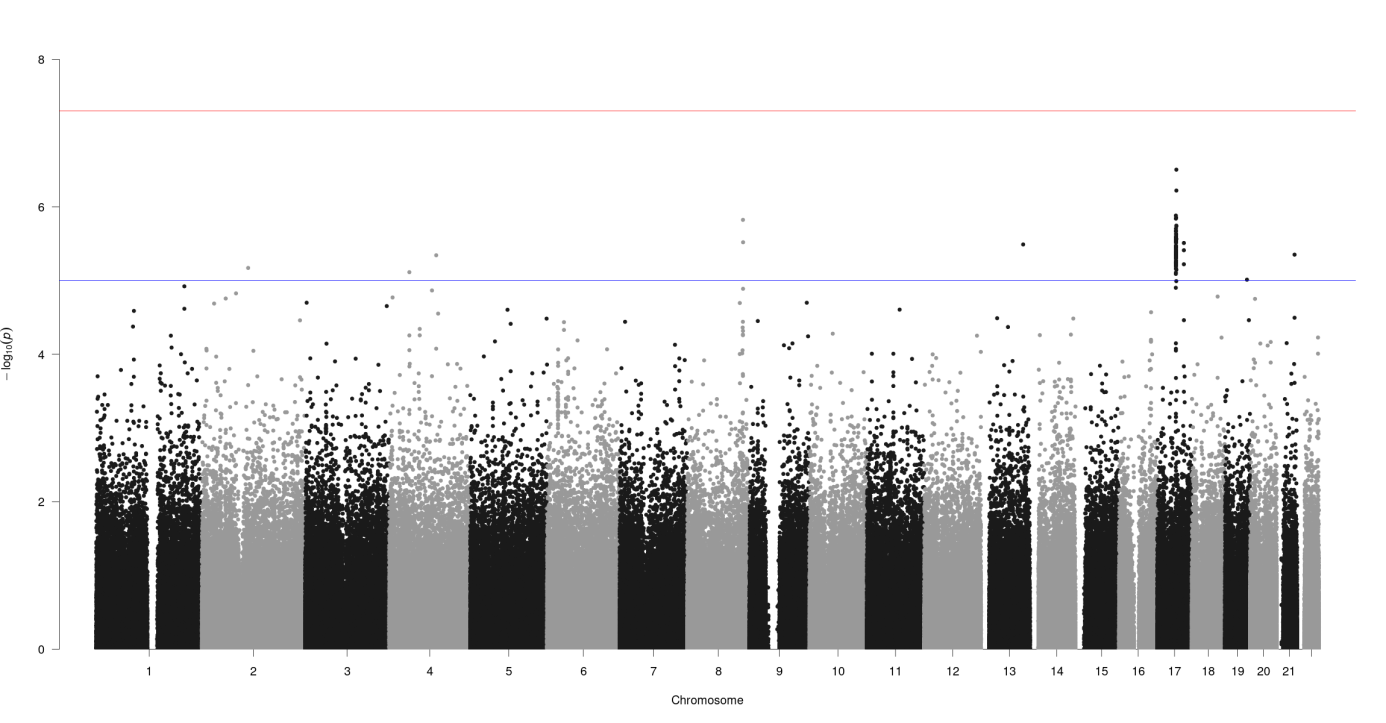

Supplement: Supplementary file 1 — Supplementary Figures. [file 41598_2021_99153_MOESM1_ESM.docx]
