# Supplementary material for: Brain age estimation at tract group level and its association with daily life measures, cardiac risk factors and genetic variants
Source: Sci Rep. 2021 Oct 18;11:20563. doi: 10.1038/s41598-021-99153-8 (PMC8523533; doi:10.1038/s41598-021-99153-8)
Supplement: Supplementary file 2 — Supplementary Table 1. [file 41598_2021_99153_MOESM2_ESM.docx]

**Table 1 –** The complete list of conditions used as exclusion criteria. ID is the Data-Field at UKB.

| **Non-cancer illness code, self-reported** | | | |
| --- | --- | --- | --- |
| **ID** | **Condition** | **ID** | **Condition** |
| 1244 | infection of nervous system | 1267 | spinal injury |
| 1245 | brain abscess/intracranial abscess | 1394 | peripheral nerve injury |
| 1246 | encephalitis | 1242 | eye/eyelid problem |
| 1247 | meningitis | 1274 | eye infection |
| 1248 | spinal abscess | 1275 | retinal problem |
| 1249 | cranial nerve problem/palsy | 1281 | retinal detachment |
| 1523 | trigemminal neuralgia | 1282 | retinal artery/vein occlusion |
| 1251 | spinal cord disorder | 1527 | retinitis pigmentosa |
| 1252 | paraplegia | 1528 | macular degeneration |
| 1524 | spina bifida | 1276 | diabetic eye disease |
| 1254 | peripheral nerve disorder | 1277 | glaucoma |
| 1255 | peripheral neuropathy | 1278 | cataract |
| 1256 | acute infective polyneuritis/guillain-barre syndrome | 1279 | eye trauma |
| 1257 | trapped nerve/compressed nerve | 1435 | optic neuritis |
| 1468 | diabetic neuropathy/ulcers | 1529 | dry eyes |
| 258 | chronic/degenerative neurological problem | 1530 | iritis |
| 1259 | motor neurone disease | 1613 | blepharitis / eyelid infection |
| 1260 | myasthenia gravis | 1243 | psychological/psychiatric problem |
| 1261 | multiple sclerosis | 286 | depression |
| 1262 | parkinsons disease | 1531 | post-natal depression |
| 1263 | dementia/alzheimers/cognitive impairment | 1287 | anxiety/panic attacks |
| 1397 | other demyelinating disease (not multiple sclerosis) | 1288 | nervous breakdown |
| 1264 | epilepsy | 1289 | schizophrenia |
| 1265 | migraine | 1290 | deliberate self-harm/suicide attempt |
| 1433 | cerebral palsy | 1291 | mania/bipolar disorder/manic depression |
| 1434 | other neurological problem | 408 | alcohol dependency |
| 1436 | headaches (not migraine) | 1409 | opioid dependency |
| 1437 | myasthenia gravis | 1410 | other substance abuse/dependency |
| 1525 | benign / essential tremor | 1469 | post-traumatic stress disorder |
| 1526 | polio / poliomyelitis | 1470 | anorexia/bulimia/other eating disorder |
| 1659 | meningioma / benign meningeal tumour | 1614 | stress |
| 1683 | benign neuroma | 1615 | obsessive compulsive disorder (ocd) |
| 1240 | neurological injury/trauma | 1616 | insomnia |
| 1266 | head injury | 1267 | spinal injury |
| **ICD10** | | | |
| G000 | G00.0 Haemophilus meningitis | I688 | I68.8 Other cerebrovascular disorders in diseases classified elsewhere |
| G001 | G00.1 Pneumococcal meningitis | G20 | G20 Parkinson's disease |
| G002 | G00.2 Streptococcal meningitis | G21 | G21 Secondary Parkinsonism |
| G003 | G00.3 Staphylococcal meningitis | G210 | G21.0 Malignant neuroleptic syndrome |
| G008 | G00.8 Other bacterial meningitis | G211 | G21.1 Other drug-induced secondary Parkinsonism |
| G009 | G00.9 Bacterial meningitis, unspecified | G212 | G21.2 Secondary Parkinsonism due to other external agents |
| G01 | G01 Meningitis in bacterial diseases classified elsewhere | G213 | G21.3 Postencephalitic Parkinsonism |
| G020 | G02.0 Meningitis in viral diseases classified elsewhere | G214 | G21.4 Vascular parkinsonism |
| G021 | G02.1 Meningitis in mycoses | G218 | G21.8 Other secondary Parkinsonism |
| G030 | G03.0 Nonpyogenic meningitis | G219 | G21.9 Secondary Parkinsonism, unspecified |
| G031 | G03.1 Chronic meningitis | G22 | G22 Parkinsonism in diseases classified elsewhere |
| G032 | G03.2 Benign recurrent meningitis [Mollaret] | G300 | G30.0 Alzheimer's disease with early onset |
| G038 | G03.8 Meningitis due to other specified causes | G301 | G30.1 Alzheimer's disease with late onset |
| G039 | G03.9 Meningitis, unspecified | G308 | G30.8 Other Alzheimer's disease |
| G040 | G04.0 Acute disseminated encephalitis | F000 | F00.0 Dementia in Alzheimer's disease with early onset |
| G042 | G04.2 Bacterial meningoencephalitis and meningomyelitis, not elsewhere classified | F001 | F00.1 Dementia in Alzheimer's disease with late onset |
| G048 | G04.8 Other encephalitis, myelitis and encephalomyelitis | F002 | F00.2 Dementia in Alzheimer's disease, atypical or mixed type |
| G049 | G04.9 Encephalitis, myelitis and encephalomyelitis, unspecified | F009 | F00.9 Dementia in Alzheimer's disease, unspecified |
| G050 | G05.0 Encephalitis, myelitis and encephalomyelitis in bacterial diseases classified elsewhere | G310 | G31.0 Circumscribed brain atrophy |
| G051 | G05.1 Encephalitis, myelitis and encephalomyelitis in viral diseases classified elsewhere | G311 | G31.1 Senile degeneration of brain, not elsewhere classified |
| G052 | G05.2 Encephalitis, myelitis and encephalomyelitis in other infectious and parasitic diseases classified elsewhere | G312 | G31.2 Degeneration of nervous system due to alcohol |
| G058 | G05.8 Encephalitis, myelitis and encephalomyelitis in other diseases classified elsewhere | G318 | G31.8 Other specified degenerative diseases of nervous system |
| G06 | G06 Intracranial and intraspinal abscess and granuloma | G319 | G31.9 Degenerative disease of nervous system, unspecified |
| G060 | G06.0 Intracranial abscess and granuloma | G320 | G32.0 Subacute combined degeneration of spinal cord in diseases classified elsewhere |
| G061 | G06.1 Intraspinal abscess and granuloma | G328 | G32.8 Other specified degenerative disorders of nervous system in diseases classified elsewhere |
| G062 | G06.2 Extradural and subdural abscess, unspecified | G230 | G23.0 Hallervorden-Spatz disease |
| G07 | G07 Intracranial and intraspinal abscess and granuloma in diseases classified elsewhere | G231 | G23.1 Progressive supranuclear ophthalmoplegia [Steele-Richardson-Olszewski] |
| G08 | G08 Intracranial and intraspinal phlebitis and thrombophlebitis | G232 | G23.2 Striatonigral degeneration |
| G09 | G09 Sequelae of inflammatory diseases of central nervous system | G233 | G23.3 Multiple system atrophy, cerebellar type |
| G35 | G35 Multiple sclerosis | G238 | G23.8 Other specified degenerative diseases of basal ganglia |
| G360 | G36.0 Neuromyelitis optica [Devic] | G239 | G23.9 Degenerative disease of basal ganglia, unspecified |
| G368 | G36.8 Other specified acute disseminated demyelination | G240 | G24.0 Drug-induced dystonia |
| G369 | G36.9 Acute disseminated demyelination, unspecified | G241 | G24.1 Idiopathic familial dystonia |
| G370 | G37.0 Diffuse sclerosis | G242 | G24.2 Idiopathic nonfamilial dystonia |
| G371 | G37.1 Central demyelination of corpus callosum | G248 | G24.8 Other dystonia |
| G372 | G37.2 Central pontine myelinolysis | G249 | G24.9 Dystonia, unspecified |
| G373 | G37.3 Acute transverse myelitis in demyelinating disease of central nervous system | G253 | G25.3 Myoclonus |
| G374 | G37.4 Subacute necrotising myelitis | G254 | G25.4 Drug-induced chorea |
| G378 | G37.8 Other specified demyelinating diseases of central nervous system | G255 | G25.5 Other chorea |
| G379 | G37.9 Demyelinating disease of central nervous system, unspecified | G258 | G25.8 Other specified extrapyramidal and movement disorders |
| G400 | G40.0 Localisation-related (focal) (partial) idiopathic epilepsy and epileptic syndromes with seizures of localised onset | G259 | G25.9 Extrapyramidal and movement disorder, unspecified |
| G401 | G40.1 Localisation-related (focal) (partial) symptomatic epilepsy and epileptic syndromes with simple partial seizures | F010 | F01.0 Vascular dementia of acute onset |
| G402 | G40.2 Localisation-related (focal) (partial) symptomatic epilepsy and epileptic syndromes with complex partial seizures | F011 | F01.1 Multi-infarct dementia |
| G403 | G40.3 Generalised idiopathic epilepsy and epileptic syndromes | F012 | F01.2 Subcortical vascular dementia |
| G404 | G40.4 Other generalised epilepsy and epileptic syndromes | F013 | F01.3 Mixed cortical and subcortical vascular dementia |
| G405 | G40.5 Special epileptic syndromes | F018 | F01.8 Other vascular dementia |
| G406 | G40.6 Grand mal seizures, unspecified (with or without petit mal) | F019 | F01.9 Vascular dementia, unspecified |
| G407 | G40.7 Petit mal, unspecified, without grand mal seizures | F020 | F02.0 Dementia in Pick's disease |
| G408 | G40.8 Other epilepsy | F021 | F02.1 Dementia in Creutzfeldt-Jakob disease |
| G409 | G40.9 Epilepsy, unspecified | F022 | F02.2 Dementia in Huntington's disease |
| G410 | G41.0 Grand mal status epilepticus | F023 | F02.3 Dementia in Parkinson's disease |
| G411 | G41.1 Petit mal status epilepticus | F024 | F02.4 Dementia in human immunodeficiency virus [HIV] disease |
| G412 | G41.2 Complex partial status epilepticus | F028 | F02.8 Dementia in other specified diseases classified elsewhere |
| G418 | G41.8 Other status epilepticus | F03 | F03 Unspecified dementia |
| G419 | G41.9 Status epilepticus, unspecified | F04 | F04 Organic amnesic syndrome, not induced by alcohol and other psychoactive substances |
| G450 | G45.0 Vertebro-basilar artery syndrome | G10 | G10 Huntington's disease |
| G451 | G45.1 Carotid artery syndrome (hemispheric) | G110 | G11.0 Congenital nonprogressive ataxia |
| G453 | G45.3 Amaurosis fugax | G111 | G11.1 Early-onset cerebellar ataxia |
| G454 | G45.4 Transient global amnesia | G112 | G11.2 Late-onset cerebellar ataxia |
| G458 | G45.8 Other transient cerebral ischaemic attacks and related syndromes | G113 | G11.3 Cerebellar ataxia with defective DNA repair |
| G459 | G45.9 Transient cerebral ischaemic attack, unspecified | G114 | G11.4 Hereditary spastic paraplegia |
| G700 | G70.0 Myasthenia gravis | G118 | G11.8 Other hereditary ataxias |
| G702 | G70.2 Congenital and developmental myasthenia | G119 | G11.9 Hereditary ataxia, unspecified |
| G708 | G70.8 Other specified myoneural disorders | G120 | G12.0 Infantile spinal muscular atrophy, type I [Werdnig-Hoffman] |
| G709 | G70.9 Myoneural disorder, unspecified | G121 | G12.1 Other inherited spinal muscular atrophy |
| G800 | G80.0 Spastic cerebral palsy | G122 | G12.2 Motor neuron disease |
| G801 | G80.1 Spastic diplegia | G128 | G12.8 Other spinal muscular atrophies and related syndromes |
| G802 | G80.2 Infantile hemiplegia | G129 | G12.9 Spinal muscular atrophy, unspecified |
| G803 | G80.3 Dyskinetic cerebral palsy | G130 | G13.0 Paraneoplastic neuromyopathy and neuropathy |
| G808 | G80.8 Other infantile cerebral palsy | G131 | G13.1 Other systemic atrophy primarily affecting central nervous system in neoplastic disease |
| G809 | G80.9 Infantile cerebral palsy, unspecified | G138 | G13.8 Systemic atrophy primarily affecting central nervous system in other diseases classified elsewhere |
| D320 | D32.0 Cerebral meninges | F050 | F05.0 Delirium not superimposed on dementia, so described |
| D321 | D32.1 Spinal meninges | F051 | F05.1 Delirium superimposed on dementia |
| D329 | D32.9 Meninges, unspecified | F058 | F05.8 Other delirium |
| D330 | D33.0 Brain, supratentorial | F059 | F05.9 Delirium, unspecified |
| D331 | D33.1 Brain, infratentorial | F060 | F06.0 Organic hallucinosis |
| D332 | D33.2 Brain, unspecified | F062 | F06.2 Organic delusional [schizophrenia-like] disorder |
| D333 | D33.3 Cranial nerves | F063 | F06.3 Organic mood [affective] disorders |
| D334 | D33.4 Spinal cord | F064 | F06.4 Organic anxiety disorder |
| D339 | D33.9 Central nervous system, unspecified | F066 | F06.6 Organic emotionally labile [asthenic] disorder |
| G122 | G12.2 Motor neuron disease | F067 | F06.7 Mild cognitive disorder |
| S06 | S06 Intracranial injury | F068 | F06.8 Other specified mental disorders due to brain damage and dysfunction and to physical disease |
| I600 | I60.0 Subarachnoid haemorrhage from carotid siphon and bifurcation | F069 | F06.9 Unspecified mental disorder due to brain damage and dysfunction and to physical disease |
| I601 | I60.1 Subarachnoid haemorrhage from middle cerebral artery | F070 | F07.0 Organic personality disorder |
| I602 | I60.2 Subarachnoid haemorrhage from anterior communicating artery | F071 | F07.1 Postencephalitic syndrome |
| I603 | I60.3 Subarachnoid haemorrhage from posterior communicating artery | F072 | F07.2 Postconcussional syndrome |
| I604 | I60.4 Subarachnoid haemorrhage from basilar artery | F078 | F07.8 Other organic personality and behavioural disorders due to brain disease, damage and dysfunction |
| I605 | I60.5 Subarachnoid haemorrhage from vertebral artery | F079 | F07.9 Unspecified organic personality and behavioural disorder due to brain disease, damage and dysfunction |
| I606 | I60.6 Subarachnoid haemorrhage from other intracranial arteries | F09 | F09 Unspecified organic or symptomatic mental disorder |
| I607 | I60.7 Subarachnoid haemorrhage from intracranial artery, unspecified | Block F70-F79 | F70-F79 Mental retardation |
| I608 | I60.8 Other subarachnoid haemorrhage | F20 | F20 Schizophrenia |
| I609 | I60.9 Subarachnoid haemorrhage, unspecified | F21 | F21 Schizotypal disorder |
| I610 | I61.0 Intracerebral haemorrhage in hemisphere, subcortical | F42 | F42 Obsessive-compulsive disorder |
| I611 | I61.1 Intracerebral haemorrhage in hemisphere, cortical | F30 | F30 Manic episode |
| I612 | I61.2 Intracerebral haemorrhage in hemisphere, unspecified | F31 | F31 Bipolar affective disorder |
| I613 | I61.3 Intracerebral haemorrhage in brain stem | F323 | F32.3 Severe depressive episode with psychotic symptoms |
| I614 | I61.4 Intracerebral haemorrhage in cerebellum | F333 | F33.3 Recurrent depressive disorder, current episode severe with psychotic symptoms |
| I615 | I61.5 Intracerebral haemorrhage, intraventricular | F50 | F50 Eating disorders |
| I616 | I61.6 Intracerebral haemorrhage, multiple localised | Block Q00-Q07 | Q00-Q07 Congenital malformations of the nervous system |
| I618 | I61.8 Other intracerebral haemorrhage | G91 | G91 Hydrocephalus |
| I619 | I61.9 Intracerebral haemorrhage, unspecified | G92 | G92 Toxic encephalopathy |
| I620 | I62.0 Subdural haemorrhage (acute) (nontraumatic) | G931 | G93.1 Anoxic brain damage, not elsewhere classified |
| I621 | I62.1 Nontraumatic extradural haemorrhage | G932 | G93.2 Benign intracranial hypertension |
| I629 | I62.9 Intracranial haemorrhage (nontraumatic), unspecified | G934 | G93.4 Encephalopathy, unspecified |
| I630 | I63.0 Cerebral infarction due to thrombosis of precerebral arteries | G935 | G93.5 Compression of brain |
| I631 | I63.1 Cerebral infarction due to embolism of precerebral arteries | G936 | G93.6 Cerebral oedema |
| I632 | I63.2 Cerebral infarction due to unspecified occlusion or stenosis of precerebral arteries | G94 | G94 Other disorders of brain in diseases classified elsewhere |
| I633 | I63.3 Cerebral infarction due to thrombosis of cerebral arteries | G95 | G95 Other diseases of spinal cord |
| I634 | I63.4 Cerebral infarction due to embolism of cerebral arteries | C710 | C71.0 Cerebrum, except lobes and ventricles |
| I635 | I63.5 Cerebral infarction due to unspecified occlusion or stenosis of cerebral arteries | C711 | C71.1 Frontal lobe |
| I636 | I63.6 Cerebral infarction due to cerebral venous thrombosis, nonpyogenic | C712 | C71.2 Temporal lobe |
| I638 | I63.8 Other cerebral infarction | C713 | C71.3 Parietal lobe |
| I639 | I63.9 Cerebral infarction, unspecified | C714 | C71.4 Occipital lobe |
| I64 | I64 Stroke, not specified as haemorrhage or infarction | C715 | C71.5 Cerebral ventricle |
| I672 | I67.2 Cerebral atherosclerosis | C716 | C71.6 Cerebellum |
| I673 | I67.3 Progressive vascular leukoencephalopathy | C717 | C71.7 Brain stem |
| I674 | I67.4 Hypertensive encephalopathy | C718 | C71.8 Overlapping lesion of brain |
| I675 | I67.5 Moyamoya disease | C719 | C71.9 Brain, unspecified |
| I680 | I68.0 Cerebral amyloid angiopathy | C720 | C72.0 Spinal cord |
| G410 | G41.0 Grand mal status epilepticus | I611 | I61.1 Intracerebral haemorrhage in hemisphere, cortical |
| G411 | G41.1 Petit mal status epilepticus | I612 | I61.2 Intracerebral haemorrhage in hemisphere, unspecified |
| G412 | G41.2 Complex partial status epilepticus | I613 | I61.3 Intracerebral haemorrhage in brain stem |
| G418 | G41.8 Other status epilepticus | I614 | I61.4 Intracerebral haemorrhage in cerebellum |
| G419 | G41.9 Status epilepticus, unspecified | I615 | I61.5 Intracerebral haemorrhage, intraventricular |
| G450 | G45.0 Vertebro-basilar artery syndrome | I616 | I61.6 Intracerebral haemorrhage, multiple localised |
| G451 | G45.1 Carotid artery syndrome (hemispheric) | I618 | I61.8 Other intracerebral haemorrhage |
| G453 | G45.3 Amaurosis fugax | I619 | I61.9 Intracerebral haemorrhage, unspecified |
| G454 | G45.4 Transient global amnesia | I620 | I62.0 Subdural haemorrhage (acute) (nontraumatic) |
| G458 | G45.8 Other transient cerebral ischaemic attacks and related syndromes | I621 | I62.1 Nontraumatic extradural haemorrhage |
| G459 | G45.9 Transient cerebral ischaemic attack, unspecified | I629 | I62.9 Intracranial haemorrhage (nontraumatic), unspecified |
| G700 | G70.0 Myasthenia gravis | I630 | I63.0 Cerebral infarction due to thrombosis of precerebral arteries |
| G702 | G70.2 Congenital and developmental myasthenia | I631 | I63.1 Cerebral infarction due to embolism of precerebral arteries |
| G708 | G70.8 Other specified myoneural disorders | I632 | I63.2 Cerebral infarction due to unspecified occlusion or stenosis of precerebral arteries |
| G709 | G70.9 Myoneural disorder, unspecified | I633 | I63.3 Cerebral infarction due to thrombosis of cerebral arteries |
| G800 | G80.0 Spastic cerebral palsy | I634 | I63.4 Cerebral infarction due to embolism of cerebral arteries |
| G801 | G80.1 Spastic diplegia | I635 | I63.5 Cerebral infarction due to unspecified occlusion or stenosis of cerebral arteries |
| G802 | G80.2 Infantile hemiplegia | I636 | I63.6 Cerebral infarction due to cerebral venous thrombosis, nonpyogenic |
| G803 | G80.3 Dyskinetic cerebral palsy | I638 | I63.8 Other cerebral infarction |
| G808 | G80.8 Other infantile cerebral palsy | I639 | I63.9 Cerebral infarction, unspecified |
| G809 | G80.9 Infantile cerebral palsy, unspecified | I64 | I64 Stroke, not specified as haemorrhage or infarction |
| D320 | D32.0 Cerebral meninges | I672 | I67.2 Cerebral atherosclerosis |
| D321 | D32.1 Spinal meninges | I673 | I67.3 Progressive vascular leukoencephalopathy |
| D329 | D32.9 Meninges, unspecified | I674 | I67.4 Hypertensive encephalopathy |
| D330 | D33.0 Brain, supratentorial | I675 | I67.5 Moyamoya disease |
| D331 | D33.1 Brain, infratentorial | I680 | I68.0 Cerebral amyloid angiopathy |
| D332 | D33.2 Brain, unspecified | I605 | I60.5 Subarachnoid haemorrhage from vertebral artery |
| D333 | D33.3 Cranial nerves | I606 | I60.6 Subarachnoid haemorrhage from other intracranial arteries |
| D334 | D33.4 Spinal cord | I607 | I60.7 Subarachnoid haemorrhage from intracranial artery, unspecified |
| D339 | D33.9 Central nervous system, unspecified | I608 | I60.8 Other subarachnoid haemorrhage |
| G122 | G12.2 Motor neuron disease | I609 | I60.9 Subarachnoid haemorrhage, unspecified |
| S06 | S06 Intracranial injury | I610 | I61.0 Intracerebral haemorrhage in hemisphere, subcortical |
| I600 | I60.0 Subarachnoid haemorrhage from carotid siphon and bifurcation | I603 | I60.3 Subarachnoid haemorrhage from posterior communicating artery |
| I601 | I60.1 Subarachnoid haemorrhage from middle cerebral artery | I604 | I60.4 Subarachnoid haemorrhage from basilar artery |
| I602 | I60.2 Subarachnoid haemorrhage from anterior communicating artery |  |  |
| **Algorithmically-defined outcomes** | | | |
| 42006 | Date of stroke | 42018 | Date of all cause dementia report |
| 42008 | Date of ischaemic stroke (should be covered by 42006) | 42022 | Date of vascular dementia report |
| 42010 | Date of intracerebral haemorrhage (should be covered by 42006) | 42024 | Date of frontotemporal dementia report |
| 42012 | Date of subarachnoid haemorrhage (should be covered by 42006) | 42021 | Date of alzheimer’s disease report |
| 42030 | Date of all cause parkinsonism report |  |  |
