# Supplementary material for: Brain age estimation at tract group level and its association with daily life measures, cardiac risk factors and genetic variants
Source: Sci Rep. 2021 Oct 18;11:20563. doi: 10.1038/s41598-021-99153-8 (PMC8523533; doi:10.1038/s41598-021-99153-8)
Supplement: Supplementary file 3 — Supplementary Table 2. [file 41598_2021_99153_MOESM3_ESM.docx]

**Table 2 –** The 27 major tracts using probabilistic tractography approach and their fiber group.

| **IDPs** | **Fiber group** |
| --- | --- |
| tract acoustic radiation (left) | Projection |
| tract acoustic radiation (right) | Projection |
| anterior thalamic radiation (left) | Projection |
| anterior thalamic radiation (right) | Projection |
| tract cingulate gyrus part of cingulum (left) | Limbic |
| tract cingulate gyrus part of cingulum (right) | Limbic |
| tract parahippocampal part of cingulum (left) | Limbic |
| tract parahippocampal part of cingulum (right) | Limbic |
| tract corticospinal tract (left) | Projection |
| tract corticospinal tract (right) | Projection |
| tract forceps major | Commissural |
| tract forceps minor | Commissural |
| tract inferior fronto-occipital fasciculus (left) | Association |
| tract inferior fronto-occipital fasciculus (right) | Association |
| tract inferior longitudinal fasciculus (left) | Association |
| tract inferior longitudinal fasciculus (right) | Association |
| tract middle cerebellar peduncle | Brainstem |
| tract medial lemniscus (left) | Brainstem |
| tract medial lemniscus (right) | Brainstem |
| tract posterior thalamic radiation (left) | Projection |
| tract posterior thalamic radiation (right) | Projection |
| tract superior longitudinal fasciculus (left) | Association |
| tract superior longitudinal fasciculus (right) | Association |
| tract superior thalamic radiation (left) | Projection |
| tract superior thalamic radiation (right) | Projection |
| tract uncinate fasciculus (left) | Association |
| tract uncinate fasciculus (right) | Association |
