# Supplementary material for: Brain age estimation at tract group level and its association with daily life measures, cardiac risk factors and genetic variants
Source: Sci Rep. 2021 Oct 18;11:20563. doi: 10.1038/s41598-021-99153-8 (PMC8523533; doi:10.1038/s41598-021-99153-8)
Supplement: Supplementary file 4 — Supplementary Table 3. [file 41598_2021_99153_MOESM4_ESM.docx]

**Table 3 –** The daily life measures that are used to perform association with brain predicted age delta .

| **The measure** | **Category** |
| --- | --- |
| Duration of heavy DIY | Physical activity |
| Duration of light DIY | Physical activity |
| Duration of walk | Physical activity |
| duration of walk for pleasuer | Physical activity |
| Time spent driving | Physical activity |
| Time spent using computer | Physical activity |
| Time spent watching TV | Physical activity |
| Length of mobile phone use | Electronic device |
| Plays computer games | Electronic device |
| Sleep duration | Sleeping habits |
| Getting up in morning | Sleeping habits |
| Nap during day | Sleeping habits |
| Sleeplessness / insomnia | Sleeping habits |
| Snoring | Sleeping habits |
| Ever smoked | Smoking |
| Smoking status | Smoking |
| Alcohol drinker status | Alcohol |
| Alcohol frequency intake | Alcohol |
| Former alcohol drinker | Alcohol |
| Time spend outdoors in summer | Sun exposure |
| Time spent outdoors in winter | Sun exposure |
| Cooked vegetable intake | Diet |
| Salad / raw vegetable intake | Diet |
| Fresh fruit intake | Diet |
| Dried fruit intake | Diet |
| Oily fish intake | Diet |
| Non-oily fish intake | Diet |
| Processed meat intake | Diet |
| Poultry intake | Diet |
| Beef intake | Diet |
| Lamb/mutton intake | Diet |
| Pork intake | Diet |
| Never eat eggs, dairy, wheat, sugar | Diet |
| [Cheese intake](http://biobank.ndph.ox.ac.uk/showcase/field.cgi?id=1408) | Diet |
| [Bread intake](http://biobank.ndph.ox.ac.uk/showcase/field.cgi?id=1438) | Diet |
| [Tea intake](http://biobank.ndph.ox.ac.uk/showcase/field.cgi?id=1488) | Diet |
| [Coffee intake](http://biobank.ndph.ox.ac.uk/showcase/field.cgi?id=1498) | Diet |
| [Water intake](http://biobank.ndph.ox.ac.uk/showcase/field.cgi?id=1528) | Diet |
