# Supplementary material for: Brain age estimation at tract group level and its association with daily life measures, cardiac risk factors and genetic variants
Source: Sci Rep. 2021 Oct 18;11:20563. doi: 10.1038/s41598-021-99153-8 (PMC8523533; doi:10.1038/s41598-021-99153-8)
Supplement: Supplementary file 5 — Supplementary Table 4. [file 41598_2021_99153_MOESM5_ESM.docx]

**Table 4 –** The association of brain predicted age delta and brain phenotypes for each model sorted by p-value. IDPs refer to image-derived phenotype. For ensemble model extra letter is added to the name of the IDP to show from which fiber (model) group it is.

| **Association model** | | | | | | |
| --- | --- | --- | --- | --- | --- | --- |
| IDP | Coefficient | std err | T value | coefficient interval_S | coefficient interval_E | corrected_pvalue |
| Mean_fa | -1.6811 | 0.0241 | -69.6434 | -1.7284 | -1.6338 | 0.0000 |
| Weighted_mean_fa | -1.4280 | 0.0251 | -56.8855 | -1.4772 | -1.3788 | 0.0000 |
| Mean_md_A | 2.5560 | 0.0210 | 121.4856 | 2.5148 | 2.5972 | 0.0000 |
| Weighted_mean_md | 2.2347 | 0.0222 | 100.4946 | 2.1911 | 2.2783 | 0.0000 |
| Mean_l1_A | 2.2083 | 0.0235 | 93.9742 | 2.1623 | 2.2544 | 0.0000 |
| Weighted_mean_l1 | 2.0557 | 0.0237 | 86.6103 | 2.0091 | 2.1022 | 0.0000 |
| Mean_l2_A | 2.3050 | 0.0219 | 105.3755 | 2.2621 | 2.3479 | 0.0000 |
| Weighted_mean_l2 | 2.2003 | 0.0222 | 99.0235 | 2.1568 | 2.2439 | 0.0000 |
| Mean_l3_A | 2.4587 | 0.0213 | 115.2263 | 2.4169 | 2.5005 | 0.0000 |
| Weighted_mean_l3 | 2.1265 | 0.0226 | 94.2058 | 2.0822 | 2.1707 | 0.0000 |
| Mean_icvf_A | -2.0701 | 0.0231 | -89.7290 | -2.1154 | -2.0249 | 0.0000 |
| Weighted_mean_icvf | -1.3834 | 0.0249 | -55.6356 | -1.4321 | -1.3347 | 0.0000 |
| Mean_isovf_A | 1.6983 | 0.0243 | 69.9553 | 1.6507 | 1.7459 | 0.0000 |
| Weighted_mean_isovf | 1.7763 | 0.0240 | 73.9643 | 1.7292 | 1.8234 | 0.0000 |
| Weighted_mean_mo | -0.6364 | 0.0275 | -23.1851 | -0.6902 | -0.5826 | 0.0000 |
| Mean_mo | -0.6047 | 0.0264 | -22.8948 | -0.6565 | -0.5530 | 0.0000 |
| Mean_od | -0.1277 | 0.0272 | -4.6940 | -0.1810 | -0.0744 | 0.0000 |
| Weighted_mean_od | -0.1065 | 0.0281 | -3.7887 | -0.1616 | -0.0514 | 0.0027 |
| **Brainstem model** | | | | | | |
| IDP | Coefficient | std err | T value | coefficient interval_S | coefficient interval_E | corrected_pvalue |
| Mean_fa | -1.4347 | 0.0166 | -86.3267 | -1.4673 | -1.4021 | 0.0000 |
| Mean_mo | -1.1532 | 0.0174 | -66.1220 | -1.1874 | -1.1191 | 0.0000 |
| Mean_md | 0.7561 | 0.0185 | 40.7778 | 0.7197 | 0.7924 | 0.0000 |
| Weighted_mean_md | 1.0745 | 0.0174 | 61.6719 | 1.0404 | 1.1087 | 0.0000 |
| Weighted_mean_l1 | 1.1649 | 0.0174 | 66.8147 | 1.1307 | 1.1991 | 0.0000 |
| Mean_l2 | 1.1952 | 0.0172 | 69.3096 | 1.1614 | 1.2290 | 0.0000 |
| Weighted_mean_l2 | 0.8853 | 0.0182 | 48.6925 | 0.8497 | 0.9210 | 0.0000 |
| Mean_l3 | 1.1125 | 0.0179 | 61.9856 | 1.0774 | 1.1477 | 0.0000 |
| Weighted_mean_l3 | 0.8957 | 0.0181 | 49.3855 | 0.8601 | 0.9312 | 0.0000 |
| Mean_od | 1.2175 | 0.0170 | 71.5308 | 1.1842 | 1.2509 | 0.0000 |
| Mean_isovf | 0.9430 | 0.0178 | 52.9441 | 0.9081 | 0.9780 | 0.0000 |
| Weighted_mean_isovf | 0.9040 | 0.0181 | 49.9920 | 0.8686 | 0.9395 | 0.0000 |
| Weighted_mean_fa | -0.2521 | 0.0203 | -12.4515 | -0.2918 | -0.2125 | 0.0000 |
| Weighted_mean_od | -0.2266 | 0.0200 | -11.3071 | -0.2659 | -0.1873 | 0.0000 |
| Weighted_mean_mo | 0.2013 | 0.0198 | 10.1734 | 0.1625 | 0.2400 | 0.0000 |
| Weighted_mean_icvf | -0.1262 | 0.0200 | -6.3158 | -0.1654 | -0.0870 | 0.0000 |
| Mean_l1 | -0.0022 | 0.0193 | -0.1122 | -0.0400 | 0.0356 | 1.0000 |
| Mean_icvf | 0.0202 | 0.0203 | 0.9961 | -0.0196 | 0.0600 | 1.0000 |
| **Commissural model** | | | | | | |
| IDP | Coefficient | std err | T value | coefficient interval_S | coefficient interval_E | corrected_pvalue |
| Mean_fa | -1.7403 | 0.0243 | -71.7110 | -1.7879 | -1.6927 | 0.0000 |
| Weighted_mean_fa | -1.1361 | 0.0255 | -44.5788 | -1.1861 | -1.0862 | 0.0000 |
| Mean_md | 2.2439 | 0.0225 | 99.8921 | 2.1998 | 2.2879 | 0.0000 |
| Weighted_mean_md | 2.1749 | 0.0227 | 95.9544 | 2.1305 | 2.2194 | 0.0000 |
| Mean_l1 | 2.0197 | 0.0234 | 86.4106 | 1.9739 | 2.0655 | 0.0000 |
| Weighted_mean_l1 | 2.1660 | 0.0236 | 91.8566 | 2.1198 | 2.2122 | 0.0000 |
| Mean_l2 | 1.8096 | 0.0239 | 75.8039 | 1.7628 | 1.8564 | 0.0000 |
| Weighted_mean_l2 | 1.9965 | 0.0231 | 86.5350 | 1.9513 | 2.0417 | 0.0000 |
| Mean_l3 | 2.3152 | 0.0223 | 103.8275 | 2.2715 | 2.3589 | 0.0000 |
| Weighted_mean_l3 | 1.9872 | 0.0232 | 85.7796 | 1.9418 | 2.0326 | 0.0000 |
| Mean_icvf | -1.4558 | 0.0256 | -56.8630 | -1.5059 | -1.4056 | 0.0000 |
| Weighted_mean_icvf | -1.5483 | 0.0247 | -62.7045 | -1.5967 | -1.4999 | 0.0000 |
| Weighted_mean_od | -1.7011 | 0.0279 | -61.0182 | -1.7558 | -1.6465 | 0.0000 |
| Mean_isovf | 1.8364 | 0.0236 | 77.6768 | 1.7901 | 1.8828 | 0.0000 |
| Weighted_mean_isovf | 1.4334 | 0.0248 | 57.8090 | 1.3848 | 1.4821 | 0.0000 |
| Mean_od | -0.3689 | 0.0265 | -13.9254 | -0.4208 | -0.3170 | 0.0000 |
| Weighted_mean_mo | 0.1489 | 0.0284 | 5.2402 | 0.0932 | 0.2045 | 0.0000 |
| Mean_mo | 0.0985 | 0.0267 | 3.6871 | 0.0461 | 0.1508 | 0.0041 |
| **Limbic model** | | | | | | |
| IDP | Coefficient | std err | T value | coefficient interval_S | coefficient interval_E | corrected_pvalue |
| Mean_fa | -2.8571 | 0.0206 | -138.8892 | -2.8974 | -2.8168 | 0.0000 |
| Mean_mo | -1.9210 | 0.0248 | -77.5291 | -1.9695 | -1.8724 | 0.0000 |
| Weighted_mean_mo | -1.4861 | 0.0260 | -57.2268 | -1.5371 | -1.4352 | 0.0000 |
| Mean_md | 3.1136 | 0.0201 | 154.7092 | 3.0742 | 3.1531 | 0.0000 |
| Mean_l1 | 1.9548 | 0.0264 | 74.1213 | 1.9031 | 2.0065 | 0.0000 |
| Mean_l2 | 3.2599 | 0.0185 | 176.2969 | 3.2237 | 3.2962 | 0.0000 |
| Weighted_mean_l2 | 1.4627 | 0.0259 | 56.4058 | 1.4119 | 1.5136 | 0.0000 |
| Mean_l3 | 3.0363 | 0.0202 | 150.2191 | 2.9967 | 3.0759 | 0.0000 |
| Mean_icvf | -1.4071 | 0.0261 | -53.9439 | -1.4582 | -1.3560 | 0.0000 |
| Weighted_mean_icvf | -1.3828 | 0.0262 | -52.8570 | -1.4341 | -1.3315 | 0.0000 |
| Mean_od | 1.6430 | 0.0257 | 63.9326 | 1.5926 | 1.6934 | 0.0000 |
| Mean_isovf | 3.1784 | 0.0205 | 155.2579 | 3.1383 | 3.2185 | 0.0000 |
| Weighted_mean_fa | -0.9910 | 0.0270 | -36.6575 | -1.0440 | -0.9380 | 0.0000 |
| Weighted_mean_l3 | 0.9606 | 0.0269 | 35.6850 | 0.9079 | 1.0134 | 0.0000 |
| Weighted_mean_md | 0.9042 | 0.0268 | 33.6989 | 0.8516 | 0.9568 | 0.0000 |
| Weighted_mean_od | 0.5310 | 0.0275 | 19.2921 | 0.4771 | 0.5850 | 0.0000 |
| Weighted_mean_isovf | -0.3176 | 0.0274 | -11.5771 | -0.3714 | -0.2638 | 0.0000 |
| Weighted_mean_l1 | -0.0790 | 0.0275 | -2.8686 | -0.1330 | -0.0250 | 0.0743 |
| **Projection model** | | | | | | |
| IDP | Coefficient | std err | T value | coefficient interval_S | coefficient interval_E | corrected_pvalue |
| Mean_fa | -1.6217 | 0.0241 | -67.4044 | -1.6689 | -1.5746 | 0.0000 |
| Mean_md | 1.9715 | 0.0227 | 86.9868 | 1.9271 | 2.0159 | 0.0000 |
| Weighted_mean_md | 1.8609 | 0.0231 | 80.7287 | 1.8157 | 1.9061 | 0.0000 |
| Mean_l1 | 1.4220 | 0.0251 | 56.6829 | 1.3728 | 1.4712 | 0.0000 |
| Weighted_mean_l1 | 1.7320 | 0.0242 | 71.6298 | 1.6846 | 1.7794 | 0.0000 |
| Mean_l2 | 1.5094 | 0.0241 | 62.5543 | 1.4621 | 1.5567 | 0.0000 |
| Weighted_mean_l2 | 1.6865 | 0.0236 | 71.3470 | 1.6402 | 1.7328 | 0.0000 |
| Mean_l3 | 2.2189 | 0.0215 | 103.1246 | 2.1767 | 2.2611 | 0.0000 |
| Weighted_mean_l3 | 1.7458 | 0.0235 | 74.3703 | 1.6998 | 1.7919 | 0.0000 |
| Mean_icvf | -1.5062 | 0.0241 | -62.4418 | -1.5535 | -1.4589 | 0.0000 |
| Weighted_mean_icvf | -1.4683 | 0.0242 | -60.5818 | -1.5158 | -1.4208 | 0.0000 |
| Weighted_mean_od | -1.2296 | 0.0271 | -45.3222 | -1.2828 | -1.1764 | 0.0000 |
| Mean_isovf | 1.2492 | 0.0247 | 50.5267 | 1.2007 | 1.2976 | 0.0000 |
| Weighted_mean_isovf | 1.0264 | 0.0252 | 40.7574 | 0.9771 | 1.0758 | 0.0000 |
| Weighted_mean_fa | -0.9067 | 0.0263 | -34.4539 | -0.9582 | -0.8551 | 0.0000 |
| Mean_od | 0.3060 | 0.0269 | 11.3718 | 0.2532 | 0.3587 | 0.0000 |
| Mean_mo | 0.2305 | 0.0266 | 8.6606 | 0.1784 | 0.2827 | 0.0000 |
| Weighted_mean_mo | 0.0381 | 0.0263 | 1.4464 | -0.0135 | 0.0897 | 1.0000 |
| **Ensemble model** | | | | | | |
| IDP | Coefficient | std err | T value | coefficient interval_S | coefficient interval_E | corrected_pvalue |
| Mean_fa_A | -1.2818 | 0.0289 | -44.3711 | -1.3384 | -1.2251 | 0.0000 |
| Mean_md_A | 1.9464 | 0.0286 | 67.9942 | 1.8903 | 2.0026 | 0.0000 |
| Weighted_mean_md_A | 1.7028 | 0.0286 | 59.5416 | 1.6467 | 1.7588 | 0.0000 |
| Mean_l1_A | 1.6788 | 0.0297 | 56.4430 | 1.6205 | 1.7371 | 0.0000 |
| Weighted_mean_l1_A | 1.5664 | 0.0295 | 53.1076 | 1.5086 | 1.6242 | 0.0000 |
| Mean_l2_A | 1.7565 | 0.0285 | 61.6554 | 1.7007 | 1.8123 | 0.0000 |
| Weighted_mean_l2_A | 1.6783 | 0.0285 | 58.9661 | 1.6225 | 1.7341 | 0.0000 |
| Mean_l3_A | 1.8742 | 0.0285 | 65.6935 | 1.8183 | 1.9301 | 0.0000 |
| Weighted_mean_l3_A | 1.6186 | 0.0286 | 56.6340 | 1.5625 | 1.6746 | 0.0000 |
| Mean_icvf_A | -1.5740 | 0.0289 | -54.4710 | -1.6307 | -1.5174 | 0.0000 |
| Mean_isovf_A | 1.2948 | 0.0291 | 44.5382 | 1.2378 | 1.3518 | 0.0000 |
| Weighted_mean_isovf_A | 1.3551 | 0.0290 | 46.7276 | 1.2982 | 1.4119 | 0.0000 |
| Mean_fa_C | -1.3263 | 0.0292 | -45.4895 | -1.3835 | -1.2692 | 0.0000 |
| Mean_md_C | 1.7095 | 0.0288 | 59.2903 | 1.6529 | 1.7660 | 0.0000 |
| Weighted_mean_md_C | 1.6591 | 0.0288 | 57.6128 | 1.6026 | 1.7155 | 0.0000 |
| Mean_l1_C | 1.5370 | 0.0290 | 52.9527 | 1.4801 | 1.5939 | 0.0000 |
| Weighted_mean_l1_C | 1.6516 | 0.0297 | 55.6885 | 1.5935 | 1.7097 | 0.0000 |
| Mean_l2_C | 1.3800 | 0.0289 | 47.7020 | 1.3233 | 1.4367 | 0.0000 |
| Weighted_mean_l2_C | 1.5225 | 0.0286 | 53.1414 | 1.4663 | 1.5786 | 0.0000 |
| Mean_l3_C | 1.7640 | 0.0289 | 61.0120 | 1.7073 | 1.8207 | 0.0000 |
| Weighted_mean_l3_C | 1.5170 | 0.0287 | 52.8381 | 1.4607 | 1.5732 | 0.0000 |
| Weighted_mean_icvf_C | -1.1763 | 0.0291 | -40.3585 | -1.2334 | -1.1192 | 0.0000 |
| Weighted_mean_od_C | -1.2944 | 0.0328 | -39.4650 | -1.3587 | -1.2301 | 0.0000 |
| Mean_isovf_C | 1.4022 | 0.0288 | 48.7488 | 1.3458 | 1.4585 | 0.0000 |
| Mean_fa_L | -2.2932 | 0.0274 | -83.5811 | -2.3470 | -2.2394 | 0.0000 |
| Mean_mo_L | -1.5405 | 0.0287 | -53.6261 | -1.5968 | -1.4842 | 0.0000 |
| Weighted_mean_mo_L | -1.1897 | 0.0291 | -40.8975 | -1.2467 | -1.1327 | 0.0000 |
| Mean_md_L | 2.4990 | 0.0280 | 89.3511 | 2.4442 | 2.5538 | 0.0000 |
| Mean_l1_L | 1.5689 | 0.0304 | 51.6461 | 1.5094 | 1.6285 | 0.0000 |
| Mean_l2_L | 2.6146 | 0.0272 | 96.0507 | 2.5613 | 2.6680 | 0.0000 |
| Weighted_mean_l2_L | 1.1752 | 0.0290 | 40.5222 | 1.1183 | 1.2320 | 0.0000 |
| Mean_l3_L | 2.4386 | 0.0278 | 87.8721 | 2.3842 | 2.4930 | 0.0000 |
| Mean_icvf_L | -1.1292 | 0.0291 | -38.8429 | -1.1861 | -1.0722 | 0.0000 |
| Mean_od_L | 1.3201 | 0.0291 | 45.3979 | 1.2631 | 1.3771 | 0.0000 |
| Mean_isovf_L | 2.5501 | 0.0285 | 89.4927 | 2.4943 | 2.6060 | 0.0000 |
| Mean_fa_P | -1.2209 | 0.0291 | -41.8994 | -1.2780 | -1.1638 | 0.0000 |
| Mean_md_P | 1.4803 | 0.0287 | 51.5540 | 1.4241 | 1.5366 | 0.0000 |
| Weighted_mean_md_P | 1.3981 | 0.0288 | 48.6037 | 1.3417 | 1.4544 | 0.0000 |
| Weighted_mean_l1_P | 1.3011 | 0.0296 | 44.0211 | 1.2432 | 1.3591 | 0.0000 |
| Mean_l2_P | 1.1345 | 0.0289 | 39.1977 | 1.0777 | 1.1912 | 0.0000 |
| Weighted_mean_l2_P | 1.2659 | 0.0289 | 43.8333 | 1.2093 | 1.3225 | 0.0000 |
| Mean_l3_P | 1.6670 | 0.0284 | 58.6613 | 1.6113 | 1.7227 | 0.0000 |
| Weighted_mean_l3_P | 1.3129 | 0.0289 | 45.4847 | 1.2563 | 1.3695 | 0.0000 |
| Mean_icvf_P | -1.1301 | 0.0289 | -39.0612 | -1.1868 | -1.0734 | 0.0000 |
| Weighted_mean_icvf_L | -1.1075 | 0.0291 | -38.0359 | -1.1646 | -1.0504 | 0.0000 |
| Weighted_mean_icvf_P | -1.1016 | 0.0290 | -38.0347 | -1.1584 | -1.0448 | 0.0000 |
| Weighted_mean_isovf_C | 1.0949 | 0.0290 | 37.7663 | 1.0381 | 1.1517 | 0.0000 |
| Weighted_mean_fa_A | -1.0896 | 0.0293 | -37.1658 | -1.1470 | -1.0321 | 0.0000 |
| Mean_icvf_C | -1.1072 | 0.0299 | -37.0364 | -1.1658 | -1.0486 | 0.0000 |
| Weighted_mean_icvf_A | -1.0535 | 0.0290 | -36.3515 | -1.1103 | -0.9967 | 0.0000 |
| Mean_l1_P | 1.0662 | 0.0298 | 35.8223 | 1.0078 | 1.1245 | 0.0000 |
| Mean_isovf_P | 0.9374 | 0.0290 | 32.3109 | 0.8806 | 0.9943 | 0.0000 |
| Mean_fa_B | -0.8977 | 0.0300 | -29.9704 | -0.9564 | -0.8390 | 0.0000 |
| Weighted_mean_fa_C | -0.8676 | 0.0292 | -29.7423 | -0.9248 | -0.8104 | 0.0000 |
| Weighted_mean_od_P | -0.9177 | 0.0316 | -29.0546 | -0.9796 | -0.8557 | 0.0000 |
| Weighted_mean_fa_L | -0.7961 | 0.0295 | -26.9894 | -0.8539 | -0.7383 | 0.0000 |
| Weighted_mean_isovf_P | 0.7737 | 0.0291 | 26.5777 | 0.7166 | 0.8307 | 0.0000 |
| Weighted_mean_l3_L | 0.7733 | 0.0293 | 26.3562 | 0.7158 | 0.8309 | 0.0000 |
| Mean_od_B | 0.7595 | 0.0293 | 25.9523 | 0.7021 | 0.8169 | 0.0000 |
| Mean_l2_B | 0.7490 | 0.0294 | 25.4366 | 0.6913 | 0.8067 | 0.0000 |
| Weighted_mean_md_L | 0.7280 | 0.0292 | 24.9409 | 0.6708 | 0.7853 | 0.0000 |
| Weighted_mean_l1_B | 0.7285 | 0.0296 | 24.6498 | 0.6706 | 0.7865 | 0.0000 |
| Mean_mo_B | -0.7206 | 0.0295 | -24.4204 | -0.7784 | -0.6627 | 0.0000 |
| Mean_l3_B | 0.6962 | 0.0300 | 23.1990 | 0.6374 | 0.7550 | 0.0000 |
| Weighted_mean_md_B | 0.6721 | 0.0291 | 23.0902 | 0.6150 | 0.7291 | 0.0000 |
| Weighted_mean_fa_P | -0.6838 | 0.0302 | -22.6624 | -0.7429 | -0.6246 | 0.0000 |
| Mean_isovf_B | 0.5900 | 0.0291 | 20.2903 | 0.5330 | 0.6470 | 0.0000 |
| Weighted_mean_isovf_B | 0.5643 | 0.0293 | 19.2506 | 0.5068 | 0.6217 | 0.0000 |
| Weighted_mean_l3_B | 0.5609 | 0.0294 | 19.1073 | 0.5033 | 0.6184 | 0.0000 |
| Weighted_mean_l2_B | 0.5532 | 0.0294 | 18.8289 | 0.4956 | 0.6108 | 0.0000 |
| Mean_md_B | 0.4740 | 0.0294 | 16.0981 | 0.4163 | 0.5317 | 0.0000 |
| Weighted_mean_mo_A | -0.4849 | 0.0307 | -15.7983 | -0.5451 | -0.4247 | 0.0000 |
| Mean_mo_A | -0.4605 | 0.0295 | -15.5943 | -0.5184 | -0.4026 | 0.0000 |
| Weighted_mean_od_L | 0.4253 | 0.0296 | 14.3537 | 0.3672 | 0.4834 | 0.0000 |
| Mean_od_C | -0.2774 | 0.0294 | -9.4234 | -0.3351 | -0.2197 | 0.0000 |
| Weighted_mean_isovf_L | -0.2496 | 0.0294 | -8.4788 | -0.3073 | -0.1919 | 0.0000 |
| Mean_od_P | 0.2327 | 0.0303 | 7.6849 | 0.1734 | 0.2921 | 0.0000 |
| Mean_mo_P | 0.1702 | 0.0299 | 5.6875 | 0.1116 | 0.2289 | 0.0000 |
| Weighted_mean_fa_B | -0.1599 | 0.0309 | -5.1697 | -0.2205 | -0.0993 | 0.0000 |
| Weighted_mean_od_B | -0.1387 | 0.0306 | -4.5341 | -0.1987 | -0.0788 | 0.0005 |
| Weighted_mean_mo_B | 0.1224 | 0.0302 | 4.0569 | 0.0633 | 0.1816 | 0.0045 |
| Weighted_mean_mo_C | 0.1128 | 0.0315 | 3.5822 | 0.0511 | 0.1745 | 0.0308 |
| Mean_od_A | -0.0932 | 0.0302 | -3.0898 | -0.1523 | -0.0341 | 0.1806 |
| Weighted_mean_icvf_B | -0.0788 | 0.0304 | -2.5892 | -0.1384 | -0.0191 | 0.8666 |
| Weighted_mean_od_A | -0.0785 | 0.0312 | -2.5196 | -0.1395 | -0.0174 | 1.0000 |
| Mean_l1_B | -0.0002 | 0.0293 | -0.0065 | -0.0577 | 0.0573 | 1.0000 |
| Mean_icvf_B | 0.0106 | 0.0309 | 0.3448 | -0.0499 | 0.0711 | 1.0000 |
| Mean_mo_C | 0.0705 | 0.0296 | 2.3817 | 0.0125 | 0.1285 | 1.0000 |
| Weighted_mean_l1_L | -0.0618 | 0.0295 | -2.0965 | -0.1196 | -0.0040 | 1.0000 |
| Weighted_mean_mo_P | 0.0280 | 0.0296 | 0.9474 | -0.0299 | 0.0859 | 1.0000 |
