# Supplementary material for: Brain age estimation at tract group level and its association with daily life measures, cardiac risk factors and genetic variants
Source: Sci Rep. 2021 Oct 18;11:20563. doi: 10.1038/s41598-021-99153-8 (PMC8523533; doi:10.1038/s41598-021-99153-8)
Supplement: Supplementary file 6 — Supplementary Table 5. [file 41598_2021_99153_MOESM6_ESM.docx]

**Table 5 –** The association of cardiac risk factors and vascular measures association with brain predicted age delta sorted by p-value for each model.

| **Association model** | | | | | | |
| --- | --- | --- | --- | --- | --- | --- |
| The measure | Coefficient | std err | T value | coefficient interval_S | coefficient interval_E | corrected_pvalue |
| Hypertens | 0.3359 | 0.0287 | 11.7021 | 0.2796 | 0.3921 | 0.0000 |
| Diabetes | 0.2306 | 0.0280 | 8.2244 | 0.1756 | 0.2856 | 0.0000 |
| cmr_LVM_i | 0.2578 | 0.0353 | 7.2965 | 0.1885 | 0.3270 | 0.0000 |
| cmr_RVEDV_i | -0.1716 | 0.0325 | -5.2829 | -0.2352 | -0.1079 | 0.0000 |
| cmr_RVSV_i | -0.1294 | 0.0295 | -4.3891 | -0.1872 | -0.0716 | 0.0002 |
| cmr_RVESV_i | -0.1380 | 0.0332 | -4.1632 | -0.2030 | -0.0730 | 0.0004 |
| cmr_LVSV_i | -0.0856 | 0.0292 | -2.9354 | -0.1427 | -0.0284 | 0.0467 |
| Deprivation | 0.0774 | 0.0282 | 2.7386 | 0.0220 | 0.1327 | 0.0865 |
| BMI | 0.0059 | 0.0284 | 0.2059 | -0.0499 | 0.0616 | 1.0000 |
| BSA | 0.0210 | 0.0467 | 0.4499 | -0.0706 | 0.1127 | 1.0000 |
| IPAQ | -0.0016 | 0.0281 | -0.0565 | -0.0566 | 0.0534 | 1.0000 |
| ASI0 | 0.0002 | 0.0285 | 0.0081 | -0.0557 | 0.0562 | 1.0000 |
| cmr_LVEDV_i | -0.0556 | 0.0308 | -1.8024 | -0.1160 | 0.0049 | 1.0000 |
| cmr_LVESV_i | 0.0033 | 0.0308 | 0.1056 | -0.0571 | 0.0636 | 1.0000 |
| **Brainstem model** | | | | | | |
| The measure | Coefficient | std err | T value | coefficient interval_S | coefficient interval_E | corrected_pvalue |
| BSA | 0.3450 | 0.0338 | 10.2229 | 0.2789 | 0.4112 | 0.0000 |
| BMI | 0.2050 | 0.0206 | 9.9739 | 0.1647 | 0.2453 | 0.0000 |
| Hypertens | 0.1894 | 0.0209 | 9.0743 | 0.1485 | 0.2303 | 0.0000 |
| Diabetes | 0.1650 | 0.0203 | 8.1145 | 0.1252 | 0.2049 | 0.0000 |
| cmr_RVEDV_i | -0.1215 | 0.0236 | -5.1586 | -0.1677 | -0.0753 | 0.0000 |
| cmr_LVM_i | 0.1249 | 0.0257 | 4.8686 | 0.0746 | 0.1752 | 0.0000 |
| cmr_RVESV_i | -0.1013 | 0.0241 | -4.2060 | -0.1485 | -0.0541 | 0.0004 |
| cmr_RVSV_i | -0.0890 | 0.0214 | -4.1617 | -0.1309 | -0.0471 | 0.0004 |
| cmr_LVSV_i | -0.0777 | 0.0211 | -3.6762 | -0.1191 | -0.0363 | 0.0033 |
| IPAQ | -0.0519 | 0.0203 | -2.5523 | -0.0918 | -0.0120 | 0.1500 |
| Deprivation | 0.0502 | 0.0205 | 2.4487 | 0.0100 | 0.0903 | 0.2009 |
| ASI0 | 0.0114 | 0.0207 | 0.5515 | -0.0292 | 0.0520 | 1.0000 |
| cmr_LVEDV_i | -0.0376 | 0.0224 | -1.6817 | -0.0815 | 0.0062 | 1.0000 |
| cmr_LVESV_i | 0.0240 | 0.0224 | 1.0718 | -0.0199 | 0.0678 | 1.0000 |
| **Commissural model** | | | | | | |
| The measure | Coefficient | std err | T value | coefficient interval_S | coefficient interval_E | corrected_pvalue |
| Hypertens | 0.2487 | 0.0286 | 8.6859 | 0.1926 | 0.3048 | 0.0000 |
| cmr_LVM_i | 0.2727 | 0.0351 | 7.7607 | 0.2039 | 0.3416 | 0.0000 |
| Diabetes | 0.1971 | 0.0279 | 7.0617 | 0.1424 | 0.2518 | 0.0000 |
| cmr_RVEDV_i | -0.1615 | 0.0323 | -4.9948 | -0.2248 | -0.0981 | 0.0000 |
| cmr_RVSV_i | -0.1194 | 0.0293 | -4.0695 | -0.1769 | -0.0619 | 0.0007 |
| cmr_RVESV_i | -0.1326 | 0.0330 | -4.0214 | -0.1973 | -0.0680 | 0.0008 |
| cmr_LVSV_i | -0.0967 | 0.0290 | -3.3331 | -0.1536 | -0.0398 | 0.0121 |
| BMI | 0.0553 | 0.0283 | 1.9554 | -0.0001 | 0.1107 | 0.7079 |
| Deprivation | 0.0541 | 0.0281 | 1.9246 | -0.0010 | 0.1091 | 0.7602 |
| BSA | 0.0649 | 0.0466 | 1.3946 | -0.0263 | 0.1562 | 1.0000 |
| IPAQ | -0.0095 | 0.0279 | -0.3404 | -0.0642 | 0.0452 | 1.0000 |
| ASI0 | -0.0062 | 0.0284 | -0.2186 | -0.0618 | 0.0494 | 1.0000 |
| cmr_LVEDV_i | -0.0469 | 0.0307 | -1.5279 | -0.1071 | 0.0133 | 1.0000 |
| cmr_LVESV_i | 0.0293 | 0.0306 | 0.9545 | -0.0308 | 0.0893 | 1.0000 |
| **Limbic model** | | | | | | |
| The measure | Coefficient | std err | T value | coefficient interval_S | coefficient interval_E | corrected_pvalue |
| Diabetes | 0.3585 | 0.0288 | 12.4471 | 0.3020 | 0.4149 | 0.0000 |
| Hypertens | 0.2934 | 0.0296 | 9.9051 | 0.2353 | 0.3514 | 0.0000 |
| BMI | 0.2599 | 0.0292 | 8.8982 | 0.2026 | 0.3171 | 0.0000 |
| cmr_RVEDV_i | -0.2732 | 0.0334 | -8.1715 | -0.3388 | -0.2077 | 0.0000 |
| BSA | 0.3831 | 0.0482 | 7.9449 | 0.2886 | 0.4776 | 0.0000 |
| cmr_RVESV_i | -0.2587 | 0.0341 | -7.5807 | -0.3256 | -0.1918 | 0.0000 |
| cmr_RVSV_i | -0.1731 | 0.0304 | -5.6969 | -0.2327 | -0.1135 | 0.0000 |
| cmr_LVEDV_i | -0.1374 | 0.0318 | -4.3263 | -0.1996 | -0.0751 | 0.0002 |
| cmr_LVSV_i | -0.1222 | 0.0301 | -4.0658 | -0.1811 | -0.0633 | 0.0007 |
| cmr_LVM_i | 0.1354 | 0.0365 | 3.7142 | 0.0639 | 0.2068 | 0.0029 |
| cmr_LVESV_i | -0.0885 | 0.0317 | -2.7907 | -0.1506 | -0.0263 | 0.0737 |
| Deprivation | 0.0577 | 0.0291 | 1.9807 | 0.0006 | 0.1147 | 0.6670 |
| IPAQ | 0.0098 | 0.0289 | 0.3376 | -0.0469 | 0.0664 | 1.0000 |
| ASI0 | -0.0066 | 0.0294 | -0.2242 | -0.0643 | 0.0511 | 1.0000 |
| **Projection model** | | | | | | |
| The measure | Coefficient | std err | T value | coefficient interval_S | coefficient interval_E | corrected_pvalue |
| Hypertens | 0.3274 | 0.0283 | 11.5799 | 0.2720 | 0.3828 | 0.0000 |
| Diabetes | 0.2146 | 0.0276 | 7.7736 | 0.1605 | 0.2687 | 0.0000 |
| cmr_LVM_i | 0.2652 | 0.0348 | 7.6176 | 0.1969 | 0.3334 | 0.0000 |
| cmr_RVEDV_i | -0.1776 | 0.0320 | -5.5550 | -0.2403 | -0.1149 | 0.0000 |
| cmr_RVESV_i | -0.1491 | 0.0327 | -4.5599 | -0.2132 | -0.0850 | 0.0001 |
| cmr_RVSV_i | -0.1291 | 0.0290 | -4.4494 | -0.1860 | -0.0722 | 0.0001 |
| cmr_LVSV_i | -0.1040 | 0.0287 | -3.6232 | -0.1603 | -0.0478 | 0.0041 |
| Deprivation | 0.0975 | 0.0278 | 3.5064 | 0.0430 | 0.1520 | 0.0064 |
| BMI | 0.0901 | 0.0280 | 3.2180 | 0.0352 | 0.1450 | 0.0181 |
| BSA | 0.1013 | 0.0463 | 2.1887 | 0.0106 | 0.1920 | 0.4009 |
| IPAQ | -0.0128 | 0.0276 | -0.4624 | -0.0669 | 0.0414 | 1.0000 |
| ASI0 | -0.0149 | 0.0281 | -0.5295 | -0.0699 | 0.0402 | 1.0000 |
| cmr_LVEDV_i | -0.0397 | 0.0304 | -1.3045 | -0.0992 | 0.0199 | 1.0000 |
| cmr_LVESV_i | 0.0491 | 0.0303 | 1.6174 | -0.0104 | 0.1086 | 1.0000 |
| **Ensemble model** | | | | | | |
| The measure | Coefficient | std err | T value | coefficient interval_S | coefficient interval_E | corrected_pvalue |
| Hypertens | 0.3074 | 0.0318 | 9.6783 | 0.2451 | 0.3697 | 0.0000 |
| Diabetes | 0.2765 | 0.0309 | 8.9359 | 0.2159 | 0.3372 | 0.0000 |
| BSA | 0.3945 | 0.0516 | 7.6500 | 0.2934 | 0.4956 | 0.0000 |
| BMI | 0.2343 | 0.0313 | 7.4784 | 0.1729 | 0.2958 | 0.0000 |
| cmr_LVM_i | 0.2752 | 0.0391 | 7.0461 | 0.1986 | 0.3517 | 0.0000 |
| cmr_RVEDV_i | -0.2052 | 0.0359 | -5.7218 | -0.2755 | -0.1349 | 0.0000 |
| cmr_RVESV_i | -0.1900 | 0.0366 | -5.1909 | -0.2617 | -0.1182 | 0.0000 |
| cmr_RVSV_i | -0.1337 | 0.0326 | -4.1047 | -0.1975 | -0.0698 | 0.0006 |
| Deprivation | 0.0963 | 0.0312 | 3.0859 | 0.0351 | 0.1575 | 0.0285 |
| cmr_LVSV_i | -0.0918 | 0.0322 | -2.8523 | -0.1549 | -0.0287 | 0.0609 |
| cmr_LVEDV_i | -0.0653 | 0.0340 | -1.9165 | -0.1320 | 0.0015 | 0.7744 |
| IPAQ | 0.0012 | 0.0310 | 0.0389 | -0.0595 | 0.0619 | 1.0000 |
| ASI0 | -0.0093 | 0.0315 | -0.2962 | -0.0711 | 0.0524 | 1.0000 |
| cmr_LVESV_i | -0.0054 | 0.0340 | -0.1600 | -0.0721 | 0.0612 | 1.0000 |
