# Supplementary material for: Brain age estimation at tract group level and its association with daily life measures, cardiac risk factors and genetic variants
Source: Sci Rep. 2021 Oct 18;11:20563. doi: 10.1038/s41598-021-99153-8 (PMC8523533; doi:10.1038/s41598-021-99153-8)
Supplement: Supplementary file 7 — Supplementary Table 6. [file 41598_2021_99153_MOESM7_ESM.docx]

**Table 6 –** The association of brain predicted age delta and daily life measures for each model.

| **Association model** | | | | | | |
| --- | --- | --- | --- | --- | --- | --- |
| The measure | Coefficient | std err | T value | coefficient interval_S | coefficient interval_E | corrected_pvalue |
| Smoking status | 0.2056 | 0.0228 | 9.0046 | 0.1608 | 0.2503 | 0.0000 |
| Ever smoked | 0.1522 | 0.0229 | 6.6426 | 0.1073 | 0.1971 | 0.0000 |
| Alcohol frequency intake | -0.1296 | 0.0232 | -5.5763 | -0.1752 | -0.0840 | 0.0000 |
| Lamb/mutton intake | 0.1017 | 0.0230 | 4.4133 | 0.0565 | 0.1469 | 0.0004 |
| Time spent watching TV | -0.0955 | 0.0231 | -4.1300 | -0.1408 | -0.0502 | 0.0014 |
| Oily fish intake | 0.0921 | 0.0232 | 3.9781 | 0.0467 | 0.1375 | 0.0027 |
| Pork intake | 0.0880 | 0.0230 | 3.8339 | 0.0430 | 0.1330 | 0.0048 |
| Tea intake | -0.0714 | 0.0228 | -3.1331 | -0.1161 | -0.0267 | 0.0659 |
| Sleep duration | 0.0712 | 0.0229 | 3.1097 | 0.0263 | 0.1160 | 0.0713 |
| Getting up in morning | -0.0703 | 0.0234 | -2.9975 | -0.1162 | -0.0243 | 0.1036 |
| Nap during day | 0.0626 | 0.0231 | 2.7109 | 0.0173 | 0.1079 | 0.2553 |
| Water intake | 0.0579 | 0.0230 | 2.5125 | 0.0127 | 0.1030 | 0.4560 |
| Beef intake | 0.0554 | 0.0229 | 2.4228 | 0.0106 | 0.1003 | 0.5857 |
| Former alcohol drinker | 0.0522 | 0.0228 | 2.2869 | 0.0075 | 0.0968 | 0.8441 |
| Duration of heavy DIY | 0.0334 | 0.0230 | 1.4523 | -0.0117 | 0.0784 | 1.0000 |
| Duration of light DIY | -0.0123 | 0.0229 | -0.5371 | -0.0573 | 0.0326 | 1.0000 |
| Duration of walk | -0.0268 | 0.0228 | -1.1738 | -0.0715 | 0.0179 | 1.0000 |
| duration of walk for pleasure | -0.0256 | 0.0229 | -1.1190 | -0.0705 | 0.0193 | 1.0000 |
| Time spent driving | 0.0101 | 0.0232 | 0.4355 | -0.0354 | 0.0556 | 1.0000 |
| Time spent using computer | 0.0370 | 0.0230 | 1.6076 | -0.0081 | 0.0820 | 1.0000 |
| Length of mobile phone use | -0.0237 | 0.0233 | -1.0194 | -0.0693 | 0.0219 | 1.0000 |
| Plays computer games | -0.0089 | 0.0228 | -0.3888 | -0.0537 | 0.0359 | 1.0000 |
| Sleeplessness / insomnia | -0.0231 | 0.0231 | -1.0021 | -0.0684 | 0.0221 | 1.0000 |
| Snoring | -0.0154 | 0.0228 | -0.6735 | -0.0601 | 0.0294 | 1.0000 |
| Alcohol drinker status | -0.0147 | 0.0228 | -0.6418 | -0.0594 | 0.0301 | 1.0000 |
| Time spend outdoors in summer | -0.0478 | 0.0233 | -2.0539 | -0.0934 | -0.0022 | 1.0000 |
| Time spent outdoors in winter | -0.0283 | 0.0231 | -1.2207 | -0.0736 | 0.0171 | 1.0000 |
| Cooked vegetable intake | -0.0086 | 0.0229 | -0.3755 | -0.0534 | 0.0362 | 1.0000 |
| Salad / raw vegetable intake | -0.0180 | 0.0229 | -0.7868 | -0.0629 | 0.0269 | 1.0000 |
| Fresh fruit intake | 0.0299 | 0.0230 | 1.3005 | -0.0151 | 0.0749 | 1.0000 |
| Dried fruit intake | 0.0179 | 0.0229 | 0.7842 | -0.0269 | 0.0627 | 1.0000 |
| Non-oily fish intake | -0.0078 | 0.0229 | -0.3423 | -0.0526 | 0.0370 | 1.0000 |
| Processed meat intake | 0.0310 | 0.0238 | 1.3051 | -0.0156 | 0.0776 | 1.0000 |
| Poultry intake | -0.0275 | 0.0228 | -1.2052 | -0.0723 | 0.0172 | 1.0000 |
| Never eat eggs, dairy, wheat, sugar | -0.0045 | 0.0229 | -0.1989 | -0.0494 | 0.0403 | 1.0000 |
| Cheese intake | 0.0254 | 0.0229 | 1.1068 | -0.0196 | 0.0703 | 1.0000 |
| Bread intake | 0.0015 | 0.0240 | 0.0615 | -0.0455 | 0.0485 | 1.0000 |
| Coffee intake | 0.0181 | 0.0229 | 0.7926 | -0.0267 | 0.0629 | 1.0000 |
| **Brainstem model** | | | | | | |
| The measure | Coefficient | std err | T value | coefficient interval_S | coefficient interval_E | corrected_pvalue |
| Smoking status | 0.1249 | 0.0163 | 7.6708 | 0.0930 | 0.1568 | 0.0000 |
| Ever smoked | 0.0969 | 0.0163 | 5.9387 | 0.0650 | 0.1289 | 0.0000 |
| Tea intake | -0.0567 | 0.0162 | -3.4875 | -0.0885 | -0.0248 | 0.0186 |
| Oily fish intake | 0.0560 | 0.0165 | 3.3958 | 0.0237 | 0.0884 | 0.0261 |
| Lamb/mutton intake | 0.0525 | 0.0164 | 3.2002 | 0.0203 | 0.0846 | 0.0523 |
| Water intake | 0.0519 | 0.0164 | 3.1629 | 0.0197 | 0.0841 | 0.0595 |
| Nap during day | 0.0504 | 0.0165 | 3.0616 | 0.0181 | 0.0827 | 0.0838 |
| Never eat eggs, dairy, wheat, sugar | -0.0465 | 0.0163 | -2.8554 | -0.0785 | -0.0146 | 0.1635 |
| Coffee intake | 0.0430 | 0.0163 | 2.6394 | 0.0111 | 0.0749 | 0.3159 |
| Pork intake | 0.0430 | 0.0164 | 2.6257 | 0.0109 | 0.0750 | 0.3289 |
| Plays computer games | 0.0414 | 0.0163 | 2.5444 | 0.0095 | 0.0733 | 0.4163 |
| Beef intake | 0.0386 | 0.0163 | 2.3689 | 0.0067 | 0.0705 | 0.6784 |
| Duration of heavy DIY | 0.0132 | 0.0164 | 0.8051 | -0.0189 | 0.0453 | 1.0000 |
| Duration of light DIY | 0.0115 | 0.0163 | 0.7057 | -0.0205 | 0.0436 | 1.0000 |
| Duration of walk | -0.0346 | 0.0163 | -2.1289 | -0.0665 | -0.0027 | 1.0000 |
| duration of walk for pleasure | -0.0148 | 0.0163 | -0.9089 | -0.0468 | 0.0172 | 1.0000 |
| Time spent driving | 0.0027 | 0.0165 | 0.1659 | -0.0297 | 0.0351 | 1.0000 |
| Time spent using computer | 0.0269 | 0.0164 | 1.6426 | -0.0052 | 0.0590 | 1.0000 |
| Time spent watching TV | 0.0071 | 0.0165 | 0.4292 | -0.0252 | 0.0394 | 1.0000 |
| Length of mobile phone use | 0.0034 | 0.0166 | 0.2047 | -0.0291 | 0.0358 | 1.0000 |
| Sleep duration | 0.0049 | 0.0163 | 0.2992 | -0.0271 | 0.0368 | 1.0000 |
| Getting up in morning | -0.0276 | 0.0167 | -1.6510 | -0.0604 | 0.0052 | 1.0000 |
| Sleeplessness / insomnia | 0.0144 | 0.0165 | 0.8754 | -0.0178 | 0.0466 | 1.0000 |
| Snoring | -0.0228 | 0.0163 | -1.3997 | -0.0546 | 0.0091 | 1.0000 |
| Alcohol drinker status | -0.0035 | 0.0163 | -0.2155 | -0.0354 | 0.0284 | 1.0000 |
| Former alcohol drinker | 0.0176 | 0.0163 | 1.0854 | -0.0142 | 0.0495 | 1.0000 |
| Time spend outdoors in summer | -0.0347 | 0.0166 | -2.0909 | -0.0672 | -0.0022 | 1.0000 |
| Time spent outdoors in winter | -0.0107 | 0.0165 | -0.6490 | -0.0430 | 0.0216 | 1.0000 |
| Cooked vegetable intake | 0.0324 | 0.0163 | 1.9890 | 0.0005 | 0.0643 | 1.0000 |
| Salad / raw vegetable intake | 0.0091 | 0.0163 | 0.5598 | -0.0228 | 0.0411 | 1.0000 |
| Fresh fruit intake | 0.0117 | 0.0164 | 0.7121 | -0.0204 | 0.0437 | 1.0000 |
| Dried fruit intake | -0.0178 | 0.0163 | -1.0946 | -0.0498 | 0.0141 | 1.0000 |
| Non-oily fish intake | 0.0217 | 0.0163 | 1.3292 | -0.0103 | 0.0536 | 1.0000 |
| Processed meat intake | 0.0212 | 0.0169 | 1.2532 | -0.0120 | 0.0544 | 1.0000 |
| Poultry intake | 0.0033 | 0.0163 | 0.2049 | -0.0286 | 0.0352 | 1.0000 |
| Cheese intake | 0.0159 | 0.0163 | 0.9765 | -0.0161 | 0.0480 | 1.0000 |
| Bread intake | -0.0102 | 0.0171 | -0.5964 | -0.0437 | 0.0233 | 1.0000 |
| Alcohol frequency intake | -0.0177 | 0.0166 | -1.0666 | -0.0501 | 0.0148 | 1.0000 |
| **Commissural model** | | | | | | |
| The measure | Coefficient | std err | T value | coefficient interval_S | coefficient interval_E | corrected_pvalue |
| Smoking status | 0.1971 | 0.0238 | 8.2854 | 0.1505 | 0.2438 | 0.0000 |
| Alcohol frequency intake | -0.1743 | 0.0242 | -7.2064 | -0.2217 | -0.1269 | 0.0000 |
| Ever smoked | 0.1505 | 0.0239 | 6.3088 | 0.1038 | 0.1973 | 0.0000 |
| Oily fish intake | 0.1359 | 0.0241 | 5.6346 | 0.0886 | 0.1831 | 0.0000 |
| Lamb/mutton intake | 0.0895 | 0.0240 | 3.7308 | 0.0425 | 0.1365 | 0.0073 |
| Water intake | 0.0890 | 0.0240 | 3.7075 | 0.0419 | 0.1360 | 0.0080 |
| Tea intake | -0.0766 | 0.0237 | -3.2248 | -0.1231 | -0.0300 | 0.0480 |
| Nap during day | 0.0766 | 0.0241 | 3.1801 | 0.0294 | 0.1238 | 0.0561 |
| Time spent watching TV | -0.0674 | 0.0241 | -2.7989 | -0.1147 | -0.0202 | 0.1951 |
| Pork intake | 0.0641 | 0.0239 | 2.6794 | 0.0172 | 0.1110 | 0.2806 |
| Time spent using computer | 0.0629 | 0.0240 | 2.6272 | 0.0160 | 0.1099 | 0.3275 |
| Duration of heavy DIY | 0.0610 | 0.0239 | 2.5497 | 0.0141 | 0.1079 | 0.4100 |
| Getting up in morning | -0.0613 | 0.0244 | -2.5122 | -0.1092 | -0.0135 | 0.4563 |
| Sleep duration | 0.0561 | 0.0238 | 2.3525 | 0.0094 | 0.1028 | 0.7091 |
| Plays computer games | -0.0542 | 0.0238 | -2.2772 | -0.1008 | -0.0075 | 0.8659 |
| Duration of light DIY | 0.0332 | 0.0239 | 1.3916 | -0.0136 | 0.0801 | 1.0000 |
| Duration of walk | -0.0120 | 0.0238 | -0.5052 | -0.0586 | 0.0346 | 1.0000 |
| duration of walk for pleasure | -0.0086 | 0.0239 | -0.3611 | -0.0554 | 0.0382 | 1.0000 |
| Time spent driving | 0.0176 | 0.0242 | 0.7283 | -0.0298 | 0.0650 | 1.0000 |
| Length of mobile phone use | -0.0167 | 0.0242 | -0.6877 | -0.0642 | 0.0308 | 1.0000 |
| Sleeplessness / insomnia | 0.0097 | 0.0241 | 0.4039 | -0.0374 | 0.0569 | 1.0000 |
| Snoring | -0.0264 | 0.0238 | -1.1091 | -0.0729 | 0.0202 | 1.0000 |
| Alcohol drinker status | 0.0089 | 0.0238 | 0.3751 | -0.0377 | 0.0556 | 1.0000 |
| Former alcohol drinker | 0.0319 | 0.0238 | 1.3416 | -0.0147 | 0.0785 | 1.0000 |
| Time spend outdoors in summer | -0.0299 | 0.0242 | -1.2333 | -0.0773 | 0.0176 | 1.0000 |
| Time spent outdoors in winter | 0.0086 | 0.0241 | 0.3570 | -0.0386 | 0.0559 | 1.0000 |
| Cooked vegetable intake | 0.0196 | 0.0238 | 0.8218 | -0.0271 | 0.0663 | 1.0000 |
| Salad / raw vegetable intake | -0.0390 | 0.0238 | -1.6380 | -0.0857 | 0.0077 | 1.0000 |
| Fresh fruit intake | -0.0171 | 0.0239 | -0.7156 | -0.0640 | 0.0298 | 1.0000 |
| Dried fruit intake | -0.0200 | 0.0238 | -0.8416 | -0.0667 | 0.0266 | 1.0000 |
| Non-oily fish intake | 0.0396 | 0.0238 | 1.6647 | -0.0070 | 0.0863 | 1.0000 |
| Processed meat intake | -0.0405 | 0.0248 | -1.6356 | -0.0891 | 0.0080 | 1.0000 |
| Poultry intake | -0.0336 | 0.0238 | -1.4126 | -0.0802 | 0.0130 | 1.0000 |
| Beef intake | 0.0256 | 0.0238 | 1.0756 | -0.0211 | 0.0723 | 1.0000 |
| Never eat eggs, dairy, wheat, sugar | -0.0447 | 0.0238 | -1.8738 | -0.0914 | 0.0021 | 1.0000 |
| Cheese intake | -0.0212 | 0.0239 | -0.8879 | -0.0680 | 0.0256 | 1.0000 |
| Bread intake | -0.0334 | 0.0250 | -1.3353 | -0.0824 | 0.0156 | 1.0000 |
| Coffee intake | 0.0284 | 0.0238 | 1.1915 | -0.0183 | 0.0750 | 1.0000 |
| **Limbic model** | | | | | | |
| The measure | Coefficient | std err | T value | coefficient interval_S | coefficient interval_E | corrected_pvalue |
| Smoking status | 0.2558 | 0.0241 | 10.5944 | 0.2084 | 0.3031 | 0.0000 |
| Ever smoked | 0.1840 | 0.0242 | 7.5945 | 0.1365 | 0.2315 | 0.0000 |
| Alcohol frequency intake | -0.1459 | 0.0246 | -5.9318 | -0.1941 | -0.0977 | 0.0000 |
| Time spent using computer | 0.1145 | 0.0243 | 4.7080 | 0.0668 | 0.1621 | 0.0001 |
| duration of walk for pleasure | -0.1092 | 0.0242 | -4.5051 | -0.1567 | -0.0617 | 0.0003 |
| Lamb/mutton intake | 0.1058 | 0.0244 | 4.3416 | 0.0580 | 0.1535 | 0.0005 |
| Oily fish intake | 0.0939 | 0.0245 | 3.8307 | 0.0459 | 0.1420 | 0.0049 |
| Snoring | -0.0864 | 0.0241 | -3.5788 | -0.1337 | -0.0391 | 0.0132 |
| Tea intake | -0.0844 | 0.0241 | -3.4970 | -0.1317 | -0.0371 | 0.0179 |
| Sleep duration | 0.0838 | 0.0242 | 3.4583 | 0.0363 | 0.1313 | 0.0207 |
| Never eat eggs, dairy, wheat, sugar | -0.0835 | 0.0242 | -3.4485 | -0.1309 | -0.0360 | 0.0215 |
| Former alcohol drinker | 0.0766 | 0.0241 | 3.1756 | 0.0293 | 0.1239 | 0.0569 |
| Nap during day | 0.0774 | 0.0245 | 3.1625 | 0.0294 | 0.1253 | 0.0596 |
| Pork intake | 0.0766 | 0.0243 | 3.1535 | 0.0290 | 0.1242 | 0.0614 |
| Water intake | 0.0694 | 0.0244 | 2.8494 | 0.0217 | 0.1172 | 0.1667 |
| Time spent driving | 0.0677 | 0.0246 | 2.7542 | 0.0195 | 0.1158 | 0.2239 |
| Beef intake | 0.0594 | 0.0242 | 2.4518 | 0.0119 | 0.1068 | 0.5405 |
| Getting up in morning | -0.0573 | 0.0248 | -2.3110 | -0.1060 | -0.0087 | 0.7923 |
| Duration of heavy DIY | 0.0361 | 0.0243 | 1.4866 | -0.0115 | 0.0838 | 1.0000 |
| Duration of light DIY | 0.0157 | 0.0243 | 0.6452 | -0.0319 | 0.0633 | 1.0000 |
| Duration of walk | -0.0238 | 0.0241 | -0.9867 | -0.0711 | 0.0235 | 1.0000 |
| Time spent watching TV | 0.0039 | 0.0245 | 0.1583 | -0.0441 | 0.0519 | 1.0000 |
| Length of mobile phone use | -0.0077 | 0.0246 | -0.3138 | -0.0559 | 0.0405 | 1.0000 |
| Plays computer games | 0.0021 | 0.0242 | 0.0849 | -0.0453 | 0.0495 | 1.0000 |
| Sleeplessness / insomnia | -0.0211 | 0.0245 | -0.8633 | -0.0691 | 0.0268 | 1.0000 |
| Alcohol drinker status | 0.0143 | 0.0242 | 0.5916 | -0.0331 | 0.0617 | 1.0000 |
| Time spend outdoors in summer | -0.0001 | 0.0246 | -0.0045 | -0.0484 | 0.0482 | 1.0000 |
| Time spent outdoors in winter | -0.0240 | 0.0245 | -0.9797 | -0.0721 | 0.0240 | 1.0000 |
| Cooked vegetable intake | 0.0093 | 0.0242 | 0.3833 | -0.0382 | 0.0567 | 1.0000 |
| Salad / raw vegetable intake | -0.0092 | 0.0242 | -0.3817 | -0.0567 | 0.0382 | 1.0000 |
| Fresh fruit intake | -0.0073 | 0.0243 | -0.2987 | -0.0549 | 0.0404 | 1.0000 |
| Dried fruit intake | -0.0233 | 0.0242 | -0.9622 | -0.0707 | 0.0242 | 1.0000 |
| Non-oily fish intake | -0.0254 | 0.0242 | -1.0501 | -0.0728 | 0.0220 | 1.0000 |
| Processed meat intake | 0.0066 | 0.0251 | 0.2612 | -0.0427 | 0.0559 | 1.0000 |
| Poultry intake | 0.0251 | 0.0242 | 1.0378 | -0.0223 | 0.0725 | 1.0000 |
| Cheese intake | -0.0314 | 0.0243 | -1.2955 | -0.0790 | 0.0161 | 1.0000 |
| Bread intake | -0.0404 | 0.0254 | -1.5899 | -0.0901 | 0.0094 | 1.0000 |
| Coffee intake | 0.0304 | 0.0242 | 1.2568 | -0.0170 | 0.0778 | 1.0000 |
| **Projection model** | | | | | | |
| The measure | Coefficient | std err | T value | coefficient interval_S | coefficient interval_E | corrected_pvalue |
| Smoking status | 0.1926 | 0.0231 | 8.3357 | 0.1473 | 0.2379 | 0.0000 |
| Ever smoked | 0.1170 | 0.0232 | 5.0469 | 0.0716 | 0.1625 | 0.0000 |
| Oily fish intake | 0.1108 | 0.0234 | 4.7290 | 0.0649 | 0.1567 | 0.0001 |
| Tea intake | -0.1009 | 0.0231 | -4.3777 | -0.1461 | -0.0557 | 0.0005 |
| duration of walk for pleasure | -0.0789 | 0.0232 | -3.4039 | -0.1243 | -0.0335 | 0.0253 |
| Water intake | 0.0770 | 0.0233 | 3.3045 | 0.0313 | 0.1227 | 0.0362 |
| Nap during day | 0.0766 | 0.0234 | 3.2730 | 0.0307 | 0.1224 | 0.0405 |
| Never eat eggs, dairy, wheat, sugar | -0.0738 | 0.0231 | -3.1919 | -0.1192 | -0.0285 | 0.0538 |
| Time spent using computer | 0.0567 | 0.0233 | 2.4391 | 0.0111 | 0.1023 | 0.5600 |
| Snoring | -0.0538 | 0.0231 | -2.3324 | -0.0990 | -0.0086 | 0.7483 |
| Former alcohol drinker | 0.0532 | 0.0231 | 2.3066 | 0.0080 | 0.0984 | 0.8014 |
| Duration of heavy DIY | 0.0503 | 0.0232 | 2.1665 | 0.0048 | 0.0958 | 1.0000 |
| Duration of light DIY | -0.0177 | 0.0232 | -0.7621 | -0.0632 | 0.0278 | 1.0000 |
| Duration of walk | 0.0140 | 0.0231 | 0.6055 | -0.0313 | 0.0592 | 1.0000 |
| Time spent driving | 0.0454 | 0.0235 | 1.9330 | -0.0006 | 0.0914 | 1.0000 |
| Time spent watching TV | -0.0120 | 0.0234 | -0.5146 | -0.0579 | 0.0338 | 1.0000 |
| Length of mobile phone use | 0.0078 | 0.0235 | 0.3304 | -0.0383 | 0.0539 | 1.0000 |
| Plays computer games | 0.0020 | 0.0231 | 0.0857 | -0.0433 | 0.0473 | 1.0000 |
| Sleep duration | 0.0018 | 0.0232 | 0.0790 | -0.0436 | 0.0472 | 1.0000 |
| Getting up in morning | -0.0520 | 0.0237 | -2.1937 | -0.0985 | -0.0055 | 1.0000 |
| Sleeplessness / insomnia | -0.0157 | 0.0234 | -0.6743 | -0.0615 | 0.0300 | 1.0000 |
| Alcohol drinker status | -0.0221 | 0.0231 | -0.9573 | -0.0674 | 0.0232 | 1.0000 |
| Time spend outdoors in summer | -0.0184 | 0.0235 | -0.7829 | -0.0646 | 0.0277 | 1.0000 |
| Time spent outdoors in winter | -0.0155 | 0.0234 | -0.6631 | -0.0614 | 0.0304 | 1.0000 |
| Cooked vegetable intake | 0.0244 | 0.0231 | 1.0524 | -0.0210 | 0.0697 | 1.0000 |
| Salad / raw vegetable intake | -0.0022 | 0.0232 | -0.0943 | -0.0476 | 0.0432 | 1.0000 |
| Fresh fruit intake | 0.0001 | 0.0232 | 0.0047 | -0.0454 | 0.0457 | 1.0000 |
| Dried fruit intake | 0.0071 | 0.0231 | 0.3061 | -0.0382 | 0.0524 | 1.0000 |
| Non-oily fish intake | 0.0111 | 0.0231 | 0.4821 | -0.0342 | 0.0565 | 1.0000 |
| Processed meat intake | -0.0254 | 0.0240 | -1.0564 | -0.0725 | 0.0217 | 1.0000 |
| Poultry intake | -0.0111 | 0.0231 | -0.4793 | -0.0563 | 0.0342 | 1.0000 |
| Beef intake | 0.0077 | 0.0231 | 0.3334 | -0.0377 | 0.0531 | 1.0000 |
| Lamb/mutton intake | 0.0153 | 0.0233 | 0.6573 | -0.0304 | 0.0610 | 1.0000 |
| Pork intake | 0.0099 | 0.0232 | 0.4255 | -0.0357 | 0.0554 | 1.0000 |
| Cheese intake | -0.0340 | 0.0232 | -1.4679 | -0.0795 | 0.0114 | 1.0000 |
| Bread intake | -0.0174 | 0.0243 | -0.7171 | -0.0650 | 0.0302 | 1.0000 |
| Coffee intake | 0.0447 | 0.0231 | 1.9323 | -0.0006 | 0.0900 | 1.0000 |
| Alcohol frequency intake | -0.0490 | 0.0235 | -2.0806 | -0.0951 | -0.0028 | 1.0000 |
| **Ensemble model** | | | | | | |
| The measure | Coefficient | std err | T value | coefficient interval_S | coefficient interval_E | corrected_pvalue |
| Smoking status | 0.2488 | 0.0263 | 9.4661 | 0.1973 | 0.3004 | 0.0000 |
| Alcohol frequency intake | -0.1880 | 0.0267 | -7.0297 | -0.2404 | -0.1356 | 0.0000 |
| Ever smoked | 0.1769 | 0.0264 | 6.7097 | 0.1252 | 0.2286 | 0.0000 |
| Water intake | 0.1087 | 0.0265 | 4.1009 | 0.0567 | 0.1606 | 0.0016 |
| Tea intake | -0.1042 | 0.0263 | -3.9670 | -0.1556 | -0.0527 | 0.0028 |
| Time spent using computer | 0.1047 | 0.0265 | 3.9559 | 0.0528 | 0.1566 | 0.0029 |
| Lamb/mutton intake | 0.0929 | 0.0265 | 3.5040 | 0.0409 | 0.1449 | 0.0175 |
| Oily fish intake | 0.0906 | 0.0267 | 3.3968 | 0.0383 | 0.1428 | 0.0260 |
| Nap during day | 0.0890 | 0.0266 | 3.3439 | 0.0368 | 0.1412 | 0.0315 |
| duration of walk for pleasure | -0.0812 | 0.0264 | -3.0786 | -0.1329 | -0.0295 | 0.0792 |
| Coffee intake | 0.0782 | 0.0263 | 2.9724 | 0.0266 | 0.1298 | 0.1125 |
| Snoring | -0.0764 | 0.0263 | -2.9077 | -0.1279 | -0.0249 | 0.1386 |
| Never eat eggs, dairy, wheat, sugar | -0.0706 | 0.0263 | -2.6832 | -0.1222 | -0.0190 | 0.2774 |
| Former alcohol drinker | 0.0654 | 0.0263 | 2.4915 | 0.0140 | 0.1169 | 0.4838 |
| Sleeplessness / insomnia | -0.0645 | 0.0266 | -2.4263 | -0.1166 | -0.0124 | 0.5801 |
| Sleep duration | 0.0634 | 0.0264 | 2.4038 | 0.0117 | 0.1151 | 0.6170 |
| Duration of heavy DIY | 0.0368 | 0.0265 | 1.3904 | -0.0151 | 0.0886 | 1.0000 |
| Duration of light DIY | 0.0506 | 0.0264 | 1.9172 | -0.0011 | 0.1024 | 1.0000 |
| Duration of walk | -0.0059 | 0.0263 | -0.2247 | -0.0574 | 0.0456 | 1.0000 |
| Time spent driving | 0.0267 | 0.0267 | 1.0003 | -0.0256 | 0.0791 | 1.0000 |
| Time spent watching TV | -0.0190 | 0.0266 | -0.7134 | -0.0712 | 0.0332 | 1.0000 |
| Length of mobile phone use | -0.0154 | 0.0268 | -0.5767 | -0.0680 | 0.0371 | 1.0000 |
| Plays computer games | 0.0136 | 0.0263 | 0.5187 | -0.0379 | 0.0652 | 1.0000 |
| Getting up in morning | -0.0248 | 0.0270 | -0.9201 | -0.0778 | 0.0281 | 1.0000 |
| Alcohol drinker status | 0.0171 | 0.0263 | 0.6504 | -0.0344 | 0.0687 | 1.0000 |
| Time spend outdoors in summer | -0.0240 | 0.0268 | -0.8954 | -0.0766 | 0.0286 | 1.0000 |
| Time spent outdoors in winter | 0.0150 | 0.0267 | 0.5615 | -0.0373 | 0.0673 | 1.0000 |
| Cooked vegetable intake | 0.0163 | 0.0263 | 0.6175 | -0.0354 | 0.0679 | 1.0000 |
| Salad / raw vegetable intake | -0.0126 | 0.0264 | -0.4782 | -0.0643 | 0.0391 | 1.0000 |
| Fresh fruit intake | 0.0047 | 0.0264 | 0.1785 | -0.0471 | 0.0565 | 1.0000 |
| Dried fruit intake | -0.0454 | 0.0263 | -1.7237 | -0.0970 | 0.0062 | 1.0000 |
| Non-oily fish intake | 0.0111 | 0.0263 | 0.4205 | -0.0405 | 0.0627 | 1.0000 |
| Processed meat intake | 0.0017 | 0.0274 | 0.0626 | -0.0519 | 0.0553 | 1.0000 |
| Poultry intake | -0.0204 | 0.0263 | -0.7777 | -0.0720 | 0.0311 | 1.0000 |
| Beef intake | 0.0527 | 0.0263 | 2.0015 | 0.0011 | 0.1043 | 1.0000 |
| Pork intake | 0.0570 | 0.0264 | 2.1551 | 0.0052 | 0.1088 | 1.0000 |
| Cheese intake | 0.0149 | 0.0264 | 0.5647 | -0.0368 | 0.0666 | 1.0000 |
| Bread intake | -0.0368 | 0.0276 | -1.3340 | -0.0910 | 0.0173 | 1.0000 |
